# Lévy-like movement patterns of metastatic cancer cells revealed in microfabricated systems and implicated in vivo

**DOI:** 10.1038/s41467-018-06563-w

**Published:** 2018-10-31

**Authors:** Sabil Huda, Bettina Weigelin, Katarina Wolf, Konstantin V. Tretiakov, Konstantin Polev, Gary Wilk, Masatomo Iwasa, Fateme S. Emami, Jakub W. Narojczyk, Michal Banaszak, Siowling Soh, Didzis Pilans, Amir Vahid, Monika Makurath, Peter Friedl, Gary G. Borisy, Kristiana Kandere-Grzybowska, Bartosz A. Grzybowski

**Affiliations:** 1 Department of Chemical and Biological Engineering, Northwestern University, 2145 Sheridan Road, Evanston, IL 60208 USA; 2 Department of Cell Biology (283) RIMLS, Radboud University Medical Centre, Geert Grooteplein 28, 6525 GA Nijmegen, The Netherlands; 3 David H. Koch Center for Applied Research of Genitourinary Cancers, Department of Genitourinary Medical Oncology, The University of Texas MD Anderson Cancer Center, Houston, TX 77030 USA; 4 Institute of Molecular Physics, Polish Academy of Sciences, Smoluchowskiego 17/19, 60-179 Poznań, Poland; 5 IBS Center for Soft and Living Matter, Ulsan National Institute of Science and Technology (UNIST), 50 UNIST-gil, Ulju-gun, 689–798 South Korea; 6 Department of Biomedical Engineering, School of Life Sciences, Ulsan National Institute of Science and Technology (UNIST), 50 UNIST-gil, Ulju-gun, 689-798 South Korea; 7 Center for General Education, Aichi Institute of Technology, 1247 Yachigusa Yakusacho, Toyota, 470-0392 Japan; 8 Faculty of Physics and NanoBioMedicine Centre, Adam Mickiewicz University, Umultowska 85, 61-614 Poznań, Poland; 9 Cancer Genomics Centre Netherlands (CG.nl), Utrecht, Netherlands; 10 The Forsyth Institute, 245 First St., Cambridge, MA 02142 USA; 11 Department of Chemistry, Ulsan National Institute of Science and Technology (UNIST), 50 UNIST-gil, Ulju-gun, 689-798 South Korea

## Abstract

Metastatic cancer cells differ from their non-metastatic counterparts not only in terms of molecular composition and genetics, but also by the very strategy they employ for locomotion. Here, we analyzed large-scale statistics for cells migrating on linear microtracks to show that metastatic cancer cells follow a qualitatively different movement strategy than their non-invasive counterparts. The trajectories of metastatic cells display clusters of small steps that are interspersed with long “flights”. Such movements are characterized by heavy-tailed, truncated power law distributions of persistence times and are consistent with the Lévy walks that are also often employed by animal predators searching for scarce prey or food sources. In contrast, non-metastatic cancerous cells perform simple diffusive movements. These findings are supported by preliminary experiments with cancer cells migrating away from primary tumors in vivo. The use of chemical inhibitors targeting actin-binding proteins allows for “reprogramming” the Lévy walks into either diffusive or ballistic movements.

## Introduction

The motility of mammalian cells has been studied for decades^1,2^, and trajectories of cell movements have been quantified in various ways. Early models of cell motility were founded on the classic Langevin equation and described the movements of adherent cells^3–5^ (for description of smaller, faster, and weakly-adherent immune cells, see ref. ^6,7^) as an Ornstein–Uhlenbeck (OU) process,^8^ such that the cell’s mean square displacement, < *x*(*t*)^2^ > , is expressed as 2*nD* (*t* – *P* (1 – exp *(-t/P)*), where *n* is the dimensionality of the system, *D* is the diffusion coefficient, and *P* is the so-called persistence time. This model predicts Gaussian distribution of velocities that are exponentially correlated in time, leading to directional persistence on short time scales (*t* *<* *P*)^9^. On long time scales (*t* » *P*), the model is reduced to a random walk and predicts uniform distribution of turning angles. This formalism has been successfully applied to describe the motions—coined persistent random walks (PRWs)—of fibroblasts, lung epithelial cells, and microvessel endothelial cells^3–5^. In addition, due to apparent directional persistence, animal as well as cellular movements have frequently been described as “correlated random walks” (CRWs)^7,10,11^. In CRWs^1^, the step sizes/times are drawn from the Gaussian or other exponentially decaying distribution, and the direction of the preceding step influences the direction of the next step. Overall, both PRW and CRW models predict that cell movements are ballistic (i.e., persistent in direction or < *x*^*2*^ *>* ~ *t*^*α*^ with *α* = 2) at short times and diffusive (*α* = 1) at long time scales. Lévy walks—recently detected in T cells^6^—are different because they are superdiffusive^12^ (i.e., < *x*^*2*^ *>* ~ *t*^*α*^ with 1 < *α* < 2) at all times and are composed of sequences of many short steps interspersed with longer "flights". This pattern is conserved across all scales, in effect giving rise to fractal patterns with no characteristic scale^13^. Mathematically, Lévy walks^14,15^ are characterized by non-Gaussian, heavy-tailed, power law distribution of persistence times/step sizes, such that *P(t)* *~* *t* ^*-μ*^, where *t* is persistence time/step size or time/distance it takes to move one step between the turns and *μ* is power law (Lévy) exponent with 1 < *μ* < 3. In the absence of a characteristic scale, the overall length of a Lévy walk is determined by the longest step and the step-length variance grows over time, though it remains finite even when unbounded by biological and environmental considerations. Most biological systems are bounded/limited (e.g., cell trajectories are limited by cell cycle and environmental conditions), resulting in truncation of the power law tail^14,16–18,^ which introduces characteristic scale to the movement pattern. However, variability around the characteristic scale is very large and self-similar, which is in sharp contrast to other finite-scale movement patterns. Lévy-like movement patterns have been observed in movements of a number of multicellular animals^17–25^ and humans^26,27^, found in trace fossil trails^17^, and recently also in T cells searching for parasite-infected cells^6^, swarming bacteria^28,29^, and even molecular motors within cells^16^. The observation of Lévy walks has been attributed to the execution of an optimal search strategy for sparsely and randomly distributed resources/target sites^30–33^, though this interpretation has not been generally accepted^13,34^.

With the general applicability of each of these models still being debated,^9,35^ one outstanding and important question concerns the movement patterns of non-metastatic versus metastatic cancer cells. Although the latter are known to have higher migration velocity and, in some cases, increased directional persistence^36–39^, it remains unclear whether increased metastatic potential is reflected by a qualitative change in the overall strategy of cell locomotion.

In this work, we address this question with a material system of micropatterned lines^10,40^ on which the cells perform one-dimensional motions which (i) have been shown^41^ to mimic the motility of cells migrating in 3D better than commonly used planar substrates and (ii) are observed in metastasizing tumor cells in vivo, attaching preferentially to and moving along linear fibers (e.g., collagen fibers^36^) or along preexisting linear perimuscular or perineural “microchannels”^42,43^. Importantly, the “microtracks” we use enable unambiguous determination of persistence times—as the times that cells move “to the left” or “to the right” before reverting their direction of motion^44^—and collection of large numbers of data points such that even the low-probability events are captured. Statistical analysis of such data then reveals that while non-metastatic cancerous cells are simple diffusive movers, the metastatic ones not only move in a superdiffusive fashion (which has been shown before^45,46^ but not in the context of metastatic cells), but also display movement patterns consistent with Lévy walks^1,47^. Significantly, we also detect Lévy walks of metastatic cells migrating in preliminary in vivo studies, in which we used state-of-the-art intravital multiphoton microscopy to resolve trajectories of individual cancer cells migrating away from animal-implanted tumors. While the generality of the observed trends certainly merits additional studies (especially in vivo), the results we describe pose some intriguing questions as to why the invasive cancerous cells have developed a movement strategy that is frequently observed in multicellular animal predators and hunters^17–25^ (as well as killer/effector T cells^6^ searching for rare target cells) and, as mentioned above, is thought to correspond to an optimal search strategy for sparsely and randomly distributed resources/target sites^30–33^ (but see also^,34^). In this context, toward the end of the paper, we describe experiments in which RNA interference and chemical inhibition of actin-binding proteins can change the Lévy walking phenotype of metastatic “predators” into either unidirectional, ballistic motions, or into diffusive migrations characterizing benign or non-invasive cancerous cells.

## Results

### Lévy walks of metastatic cancer cells revealed on linear microtracks

**Fig. 1:**
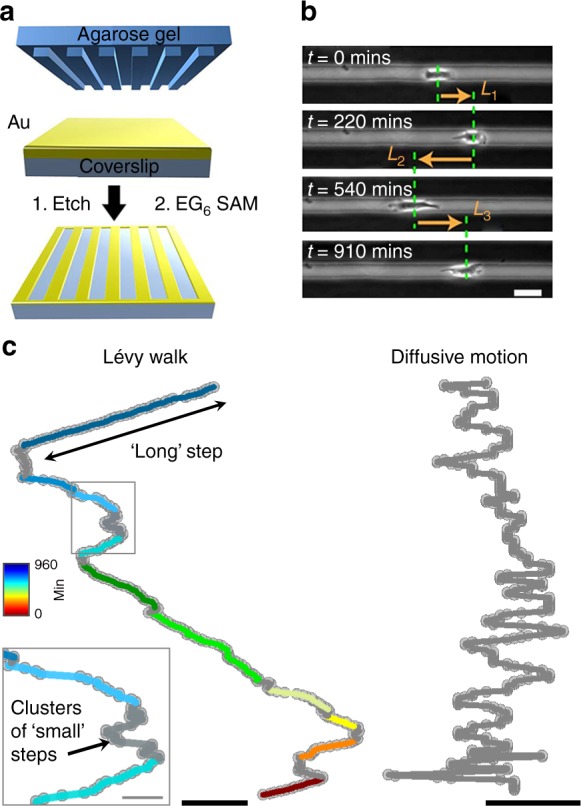
Trajectories of cancer cell motions on linear microtracks. **a** Scheme of substrate fabrication using the Wet Etching technique^40, 48, 49^. **b** Optical micrographs showing a cell migrating on a microtrack (scale bar = 20 µm). The cell moves along the etched, optically transparent microtrack, and not across cell-adhesion-resistant dark regions. The definition of step persistence length/time is the distance/time the cell travels in one direction before it reverts the direction of motion. The arrows labeled *L*_*1*_, *L*_*2*_, and *L*_*3*_ indicate three consecutive “steps” of the cell (here, to the right, to the left, and to the right again). **c** A representative trajectory of a metastatic cell comprised of clusters of “small” steps (shown in gray) interspersed with “large” steps (color denotes elapsed time and each long step is in different color) is characteristic of a Lévy walk (see also Supplementary Figure 2 for long-term trajectories). Scale bar is 100 μm for Lévy trajectory and 20 μm for the inset. This can be contrasted with a trajectory of a non-metastatic cell exhibiting diffusive motion (all steps are small and shown in gray, scale bar is 20 μm). Note that while cell motions in experiments are in 1D (along microtracks), the vertical axis in the trajectories shown here corresponds to time (from top to bottom). Total length of each trajectory is 960 min with each point 3 min apart. See also Supplementary Movies 1–6. The distinction between “small” and “large” steps is best appreciated by viewing long-term Supplementary Movies 13–15

Most of the in vitro experiments were performed on linear microtracks etched in gold-on-glass substrates using the so-called Wet Etching technique^40,48,49^ (Fig. 1a; for all fabrication details, see Supplementary Methods/Supplementary Note 1). The gold regions were protected with self-assembled monolayers, SAMs^50,51^, of oligo (ethylene glycol)-terminated alkyl thiols (HS(CH_2_)_11_(OCH_2_CH_2_)_6_OH; EG_6_ (ProChimia Surfaces, Gdansk, Poland: www.prochimia.com) known to prevent cell adhesion. The unprotected glass lines were covered with either Laminin (Sigma-Aldrich, cat. # L2020) or Laminin 5 (LN 5; extracted from 804G cells in crude form, as previously described^52^)—these extracellular matrix substrates were chosen because of their motility-promoting characteristics (vs. more adhesive fibronectin) and because they are routinely used as physiologically relevant substrates of choice for the respective cell lines.

**Fig. 2:**
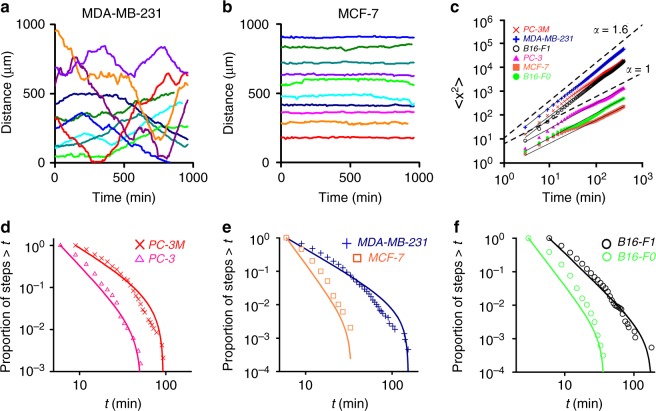
Superdiffusive and Lévy walks of metastatic cancer cells on linear microtracks. **a** Typical trajectories/displacement versus time of highly metastatic cells (here for MDA-MB-231) feature characteristic small steps interspersed with unidirectional, long excursions. **b** In contrast, trajectories of non-metastatic cells (here for MCF-7) are more random/”jiggly”. Ten representative trajectories per cell type are shown. The starting points for trajectories are randomly positioned along the y axis (“Distance”) for clarity. See also Supplementary Movies 1–6 and 13–15 and Supplementary Figure 1 for trajectories for PC-3, PC-3M, B16-F0, and B16-F1 cells and Supplementary Figure 2 for long-term trajectories. **c** Differences in the two modes of motility are quantified in the log–log plots of the cells’ mean square displacement (in μm^2^) versus time, $\left\langle {x^2} \right\rangle \propto t^\alpha$. The values of *α* close to unity (PC-3: *α* *=* 1.04, 95% confidence interval ± 0.03; MCF-7: *α* = 0.96 ± 0.04; B16F0: *α* = 1.05 ± 0.02) indicate diffusive walks of non-metastatic cells. Metastatic cells are superdiffusive (PC-3M: *α* = 1.58 ± 0.02; MD-MB-231: *α* = 1.54 ± 0.01; B16F1: *α* = 1.52 ± 0.02). **d**–**f** The cumulative frequency distributions, CFDs, of persistence times (*t*) for all types of cells studied on microtracks. Markers are experimental statistics: magneta triangles for PC-3, red crosses for PC-3M, blue crosses for MDA-MB-231, orange rectangles for MCF-7, green circles for B16-F0, and black circles for B16-F1. Solid lines are theoretical truncated power law fits. Statistical analysis of cancer cell movements on 1D microtracks is shown in Table 1

When the cells were applied (at plating density of ~10,000 cells/cm^2^) onto microstructured substrates presenting arrays of 20-μm-wide linear tracks, they localized exclusively onto these tracks, spread, and, to a good approximation, displayed one-dimensional motions (Fig. 1b). We compared and contrasted motions of six types of cells from three cancers (Fig. 2; Supplementary Figure 1): non-metastatic PC-3 and metastatic PC-3M^53^ prostate cancer cells; non-metastatic MCF-7 and metastatic MDA-MB-231^38^ breast cancer cells; and non-metastatic B16-F0 and metastatic B16-F1^54^ mouse melanoma cells. Regarding the cell line choices, we note that for B16 and PC lines, cells are termed metastatic versus non-metastatic based on, respectively, their low and high metastatic potentials^53,54^. For breast cancer lines, the MCF-7 cell line retains several characteristics of differentiated mammary epithelium and represents a poorly invasive luminal subtype of breast cancer, whereas the MDA-MB-231 line represents a highly invasive basal subtype of breast cancer^55^.

Using an automated image acquisition and analysis protocol developed in-house, we were able to monitor cell motions for up to 16 h and collect robust statistics with *n* = 17–69 different cells and ~5120–20,800 time points per every cell type studied (see Table 1 legend). Only tracks housing one cell (~60% of all tracks at the plating density used) were analyzed to eliminate any potential artifacts due to cell–cell collisions (see Supplementary Note 1 for comment on cell collisions). The typical trajectories of cells on the microtracks shown in Fig. 1c, Fig. 2a, b, and Supplementary Figure 1 are characteristic of, respectively, metastatic and non-metastatic cells (see Supplementary Movies 1 vs. 2, 3 vs. 4 and 5 vs. 6; also Supplementary Movies 13–15).

**Table 1:** Statistical analysis of cancer cell movements on 1D microtracks

Cell type	MSD (*α*)	MLE for *μ*	MLE for *μ*	Akaike weights				
		Power law	Truncated power law	TP	P	LN	SE	E
PC-3M	1.58 ± 0.02	2.39 ± 0.2, *a* = 18	**2.22 ± 0.16**, *a* = 9	**0.98**	< 0.01	< 0.01	< 0.01	< 0.01
MDA-MB-231	1.54 ± 0.01	2.58 ± 0.4, *a* = 12	**2.49 ± 0.07**, *a* = 6	**> 0.99**	< 0.01	< 0.01	< 0.01	< 0.01
B16-F1	1.52 ± 0.02	2.50 ± 0.2, *a* *=* *3*	**2.99** ± **0.09**, *a* *=* *6*	**0.57**	0.20	0.23	< 0.01	< 0.01
PC-3	1.04 ± 0.03	3.17 ± 0.5, *a* = 12	**3.14 ± 0.19**, *a* = 6	**> 0.99**	< 0.01	< 0.01	< 0.01	< 0.01
MCF-7	0.96 ± 0.04	4.57 ± 1.1, *a* = 6	**4.52 ± 0.9**, *a* = 6	**0.77**	< 0.01	< 0.22	< 0.01	< 0.01
B16-F0	1.05 ± 0.02	3.36 ± 1.3, *a* = 6	**3.69 ± 0.09**, *a* = 3	**> 0.99**	< 0.01	< 0.01	< 0.01	< 0.01

The first column shows exponent α values obtained from mean square displacement vs. time plots shown in Fig. 2c. Other columns show values for *μ* exponent – both from power law and truncated power law fits – calculated using the maximum likelihood estimates (MLEs), as described in detail in Supplementary Note 1/Supplementary Methods, and Akaike weights (*w*AIC) for all model comparisons. Parameter *a* is the lower cutoff parameter determined using reweighted Kolmogorov–Smirnov (KS) statistic as described in^56^ (see also Supplementary Methods for more details). The 95% confidence intervals (CIs) are shown for all values. For all cell types, truncated power law (TP), power law (P), log-normal (LN), stretched exponential (SE), and exponential (E) distributions were compared. Strongest supported model is highlighted in bold. *w*AIC comparisons were performed using lower cutoff values *a* (see Supplementary Tables 1 and 2) that are specified for the truncated power law. Cumulative frequency distributions (CFDs, shown in Fig. 2d–f) for metastatic cells (PC-3M, MDA-MB-231, B16-F1) fit best the truncated power law with 2 < *μ* < 3 consistent with Lévy walks (see Supplementary Figure 5 and Supplementary Methods for details on all model comparisons); non-metastatic cells (PC-3, MCF-7, B16-F0) fit best truncated power law with *μ* > 3 consistent with diffusive motion. See Supplementary Movies 1–6. Analyses were based on the following numbers of cells and time points: MDA-MB-231 (*n* *=* 69 cells, *m* = 17,569 time points*)*; MCF-7 (*n* = 15, *m* = 4,468); PC-3M (*n* = 18, *m* = 4,833); PC-3 (*n* = 17, *m* = 5,457); B16-F1 (*n* = 53, *m* = 14,640); B16-F0 (*n* = 62, *m* = 18,585)

To quantify these cell trajectories and the dynamics of cell motions, we first plotted cells’ mean square displacements, $\left\langle {x^2} \right\rangle = \left\langle {\left[ {x(t + t_0) - x(t_0)} \right]^2} \right\rangle$, versus time, *t*, where $\left\langle {...} \right\rangle$ denotes a combined average over all starting times *t*_0_ and cell paths (see refs. ^12,45^). When plotted on a log–log scale, these dependencies give straight lines corresponding to $\left\langle {x^2} \right\rangle = t^\alpha$ scaling, with the slope of the lines being the exponent *α*. From statistical physics, it is well known^12^ that *α* = 1 indicates diffusive motion, values 1 < *α* *<*2 correspond to superdiffusion, and *α* = 2 is characteristic of ballistic motion (e.g., unidirectional motion without turns). As evidenced by the plots in Fig. 2c, all non-metastatic cells we studied move diffusively (*α* *~* 1) while their highly metastatic variants move in a superdiffusive fashion (*α* *~* 1.5–1.6). These characteristics are conserved over the entire time domain and not only short term (as CRW or PRWs).

Long-term superdiffusive behavior is sometimes found in systems performing Lévy walks,^47^ which are a sub-class of random walks and are characterized by periods of small steps interspersed with long but infrequent unidirectional excursions. Typical cell trajectories such as those shown in Fig. 1c and Fig. 2a (see also Supplementary Figure 1–3 and Supplementary Movies 1, 3, 5, and 13–15) suggest that the metastatic MDA-MB-231, PC-3M, and B16-F1 cells might indeed be performing Lévy walks. This hypothesis can be mathematically verified by the analysis of cells' cumulative frequency distributions, CFDs, of persistence times (i.e., the persistence time is defined as the time that cell moves persistently in a given direction^12^). When such distributions are plotted on a log–log scale, it is evident that the CFDs corresponding to metastatic cells are more heavy-tailed than CFDs corresponding to non-metastatic ones (Fig. 2 d–f). However, extreme care must be taken when assigning such trends to a specific functional form, especially given recent findings^56,57^ that the use of inaccurate statistical methods has led to an incorrect assignment of the search/movement patterns as Lévy flights in a number of studies. Accordingly, we followed the rigorous procedure developed by Edwards et al.^57,58^ to test power law distributions using the likelihood and Akaike weights (which measure an appropriateness of a given fit^57,58^) and tested multiple alternative competing models (see also Supplementary Notes 1 and 2, and Supplementary Tables 1, 2) as detailed by Clauset et al^56^. In addition to power law and exponential models, we also considered heavy-tailed log-normal (observed in motions of T cells within lymph nodes^7^), heavy-tailed stretched exponential^56^, as well as a truncated power law^59^ (in which power law tail of Lévy walk is truncated).

In particular, the truncated power law was considered because data collection time was limited for both short and long times. The short time limitation at *~*3–5 min was due to relatively large cell sizes and small speeds (respectively, ~20–40 μm and ~0.25–2 μm/min for metastatic cells vs. *~* 7 μm and *~* 6–12 μm/min for T cells studied in ref. ^6^), making it difficult to (1) distinguish their translocation from just the membrane dynamics, and (2) limit the photo-damage imposed to cells by laser/light exposure. The long time limitation was due to the cells dividing (e.g., ~30 h for MDA-MB-231). It is therefore impossible to obtain single-cell data spanning the desirable two decades of persistence times and enhancing statistics of low-probability, long cellular excursions in the power law’s tail^60^. Instead, we (akin to others^9,35,46^) have analyzed persistence-time distributions over the populations of single cells (see also Supplementary Figure 4 for single-cell analysis). This being said, to capture as many long excursions as possible, in some experiments we managed to record individual cellular trajectories for up to 40 h (if cells divided during observation time, they were tracked only up to division event) and total displacements up to 1000 μm (see Supplementary Figures 2, 5, Supplementary Table 3, and Supplementary Movies 13–15).

The results summarized in Fig. 2d–f and Table 1 demonstrate that only the metastatic cells (PC-3M, MDA-MB-231, B16-F1) exhibit log–log distributions of persistence times that can be assigned as Lévy walks. While the cumulative frequency distribution, CFD, plots might “appear” to fit a simple power law ($P(t) = (\mu - 1)a^{\mu - 1}t^{ - \mu },t \ge a$, where $(\mu - 1)a^{\mu - 1}$ is the normalization constant and parameter *a* determines the time at which a power law fit is appropriate; *a* values are listed in Table 1), detailed maximum likelihood and Akaike-weight analyses evidence that a more appropriate fit is a truncated power law, $P\left( t \right) = \left( {\frac{{\mu - 1}}{{a^{1 - \mu } - b^{1 - \mu }}}} \right)t^{ - \mu }$, where *b* is an upper truncation parameter corresponding to the maximal point in each dataset. Importantly, the values of exponents *µ* in these fits are below 3, corresponding to Lévy walks. Moreover, such analyses confirm that the truncated power law fit is superior to all alternative models considered (Fig. 2d–f, Table 1, see also Supplementary Figure 5 for CFDs and model fits; Supplementary Figure 6 for evaluation of log-normal distribution; Supplementary Tables 1 and 2 and Supplementary Methods for all additional details on model fitting and comparisons). All data sets for metastatic cells pass the goodness-of-fit test performed according to the method detailed in ref. ^56^ (Supplementary Note 3 and Supplementary Figure 7). In this context, it is important to note that truncated power law (with *µ* *~*1–3) has become increasingly favored (over pure power law) as a more relevant model for describing bounded/limited biological systems and—similar to a pure power law—is considered Lévy walk^14,16–18^. We also note that (i) “walks” rather than “flights”^15^ terminology is appropriate since, as we verified, our migrating cells exhibit strong correlation between persistence times and persistence lengths (i.e., step sizes); and (ii) observing Lévy-like patterns in a simple 1D system in the absence of chemoattractant gradients suggests that they may be an inherent characteristic of metastatic cells.

Regarding non-metastatic cells, their CFDs also fit best to a truncated power law, but the exponents *μ* in these fits are greater than 3 (Fig. 2d–f and Table 1**)**, which is in line with the purely diffusive^34,47^ nature of these cells’ motions.

### Lévy walks of metastatic cancer cells in live tumors

**Fig. 3:**
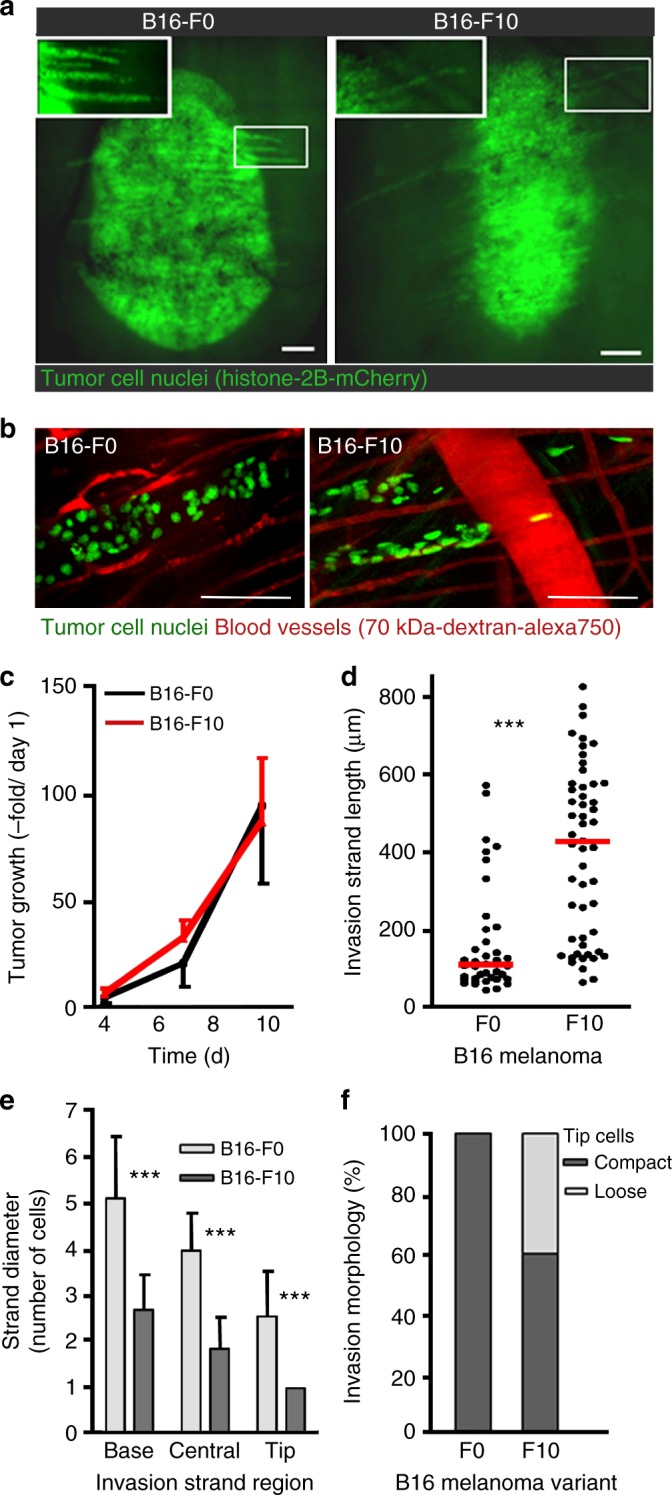
Structure of the linear invasion strands in vivo. **a** Live B16-F0 (non-metastatic) and B16-F10 (metastatic) tumors in mouse skin imaged with epifluorescence microscopy (cell nuclei marked with Histone-2B/mCherry, green). Images are representative of least six tumors from at least three independent mice per group. Insets show finger-like invasion strands moving away from the main tumor mass. Scale bar is 100 μm. **b** Enlarged images corresponding to the tips of the invasion strands. Few (~2–4) B16-F10 cells, so-called tip cells, detached from the invasion strands and exhibited trajectories that over limited observation time appeared ballistic (see also Fig. 4b, Zone 5). These tip cells were observed only in the very outer zone 5 which was not included in Lévy walk analysis because of small number of cells in this zone. Scale bar is 100 μm. Quantification of (**c**) tumor growth (quantification based on tumor volume and expressed as an increase over volume measured on day 1; time, days) and (**d**) invasion strand length on day 6. Invasion strands were statistically longer in metastatic tumors (average ~400 μm in B16-F10 vs. ~100 μm in B16-F0, red horizontal lines). Error bars in **c** are standard deviations based on ≥ 30 invasion strands from at least six tumors of three individual mice. **e** The widths of invasion strands near the base (i.e., beginning of strand at tumor’s edge), center between base and tip, and the tip region (recorded on day 6; error bars give standard deviations of ≥7 invasion strands from at least four tumors of three individual mice). **f** Individualized (with separation above two nuclear diameters) versus compact cell positioning in leading tips of invasion strands

While the microtracks offer a convenient platform for collecting large numbers of motility data, these ex vivo results are not necessarily indicative of in vivo motility patterns, which may be influenced by local chemotactic gradients and/or cell interactions with the tissue microenvironment. Extension to in vivo, however, is technically challenging^36,61^ and further complicated in our case where resolving individual cells over long (hours) times is necessary for the collection of statistics ample enough to construct reliable probability distributions. Also, it should be remembered that in vivo analyses cannot offer complete correspondence with all the cell types studied on microtracks and are limited to tumors (i) lying within the penetration depth of the existing high-resolution microscopy modalities (max. 600 μm in mouse dermis), and (ii) for which appropriate animal protocols have been validated and approved. In our study, the additional requirement to compare metastatic versus non-metastatic cells of the same origin limited the available choices to a pair of non-metastatic B16-F0 and metastatic B16-F10 mouse melanoma cells. The trajectories of these cells invading the mouse dermis were recorded using a dorsal skin-fold chamber model^43^. Using combined near-infrared and infrared multiphoton microscopy^43^, second-harmonic generation (SHG) allowed for the reconstruction of fibrillar collagen and myofibers, while fluorescence enabled visualization of blood vessels and tracking of moving cell nuclei (Fig. 3).

**Fig. 4:**
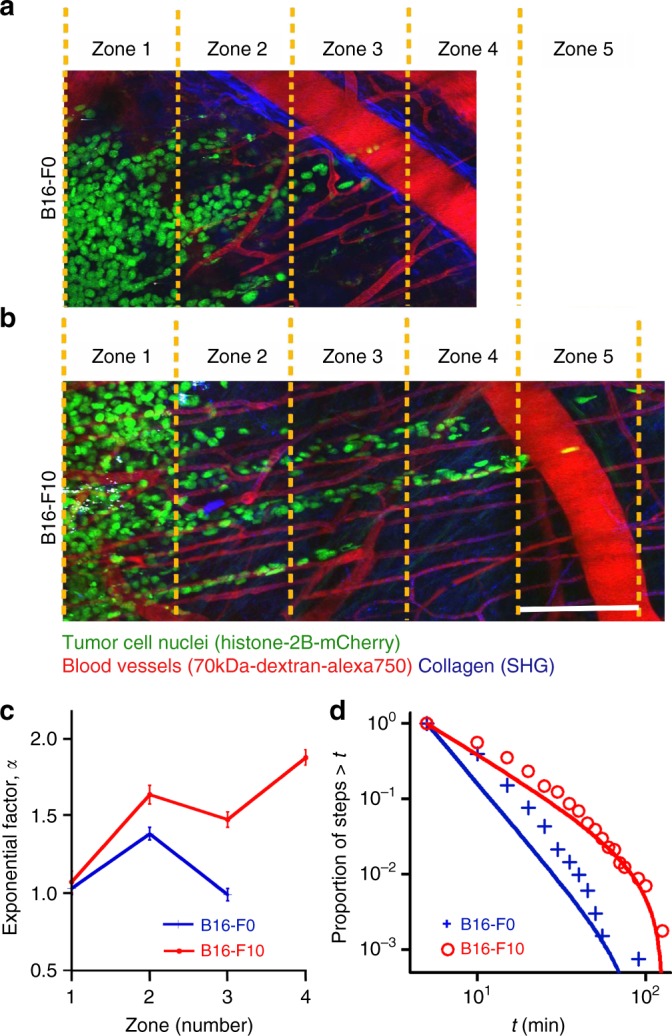
Migration of non-metastatic and metastatic cells from live tumors in mouse skin. **a**, **b** Cell movements within invasion strands formed by up to 100 tumor cells and relation to the guidance structures of the tumor microenvironment, observed by using high-resolution multiphoton microscopy (see Supplementary Movies 7 and 8 for trajectories). The snapshots from these movies are shown for (**a**) B16-F0 and (**b**) B16-F10 tumors. AlexaFluor 750-labeled dextran was used to visualize blood vessels (red), Histone-2B/mCherry marks the nuclei of cells (*green*), and fibrillar collagen was revealed by SHG (second-harmonic generation; blue). Dashed yellow lines divide the invasion strands into five zones, each 150-μm wide. Scale bar is 150 μm. **c** The values of the “diffusion exponents” *α* indicate that both B16-F0 and B16-F10 cells are diffusive at the tumor margin (zone 1, *α~*1) and superdiffusive (zone 2, *α~*1.4–1.6) with entering the invasion zone. Away from the tumor (zones 3–4), however, the metastatic cells remain superdiffusive while the non-metastatic ones show diffusive behavior. Error bars correspond to 95% confidence intervals. **d** The cumulative frequency distributions, CFDs, of persistence times are truncated power law with *μ* *>* *3* for diffusive B16-F0 (blue crosses, Zones 1–3) and truncated power law with *μ~2.36* for B16-F10 (red circles, Zones 2–4) performing Lévy walks. Markers are experimental statistics for persistence times. Solid lines are truncated power law fits. Statistical analysis of cancer cell movements in vivo is shown in Table 2

On one hand, both B16-F0 and B16-F10 cells predominantly formed invasion strands consisting of up to 100 individual cells which jointly infiltrated the dermis by following linear tissue interfaces provided by parallel perimuscular and perineural microtracks, blood vessels, or collagen bundles (Fig. 3). On the other hand, the path organization and kinetics of cell motions were markedly different for the non-metastatic (Fig. 4a and Supplementary Movie 7) and metastatic cells (Fig. 4b and Supplementary Movie 8). To quantify these motions, we divided the entire tumor/strand domain into 150-μm wide zones with zone 1 near the border of the tumor mass and incremental outward numbering along the invasion zone (see Fig. 4a, b and Table 2). The path organizations were found to be strongly zone-dependent. At the tumor margin (zone 1), where cells are “crowded” and frequent cell–cell collisions occur, the motions of both B16-F0 and B16-F10 cells are “jiggly” and purely diffusive, as evidenced by the “diffusion exponent” *α* *~* 1 (Fig. 4c). Beyond the tumor border, within zone 2, the cells experience more frequent collisions from one side (zone 1) and are effectively “pushed away” from the tumor exhibiting slightly differing degrees of superdiffusive motions (*α* *~* 1.38 for B16-F0 and *α* *~* 1.63 for B16-F10). The differences between the inherent characteristics of cell motions—i.e., not dominated by crowding effects/cell–cell collisions—manifest themselves fully in zones 3 and 4, with non-metastatic cells reverting to diffusive motions (*α* *~* 1) while metastatic cells remaining evidently superdiffusive, with *α* *~* 1.47–1.87. These characteristics were conserved over the entire time domain (i.e., not only short term as in correlated/persistent random walks) and were statistically significant as evidenced by the 95% confidence intervals for *α* listed in Table 2.

**Table 2:** Statistical analysis of cancer cell movements in vivo

Cell type	MSD (*α*)	MLE for *μ*	Akaike weights		
		Truncated power law	TP	P	E
B16-F0					
Zone 1	1.03 ± 0.04	3.58 ± 0.24	0.77	0.22	< 0.01
Zone 2	1.38 ± 0.04	3.57 ± 0.21	0.57	0.42	< 0.01
Zone 3	0.99 ± 0.04	3.85 ± 0.35	0.59	0.40	< 0.01
Zone 4	–	–	–	–	–
B16-F10					
Zone 1	1.07 ± 0.07	4.97 ± 0.55	0.58	0.41	< 0.01
Zone 2	1.63 ± 0.06	**2.34** ± **0.21**	**0.90**	0.09	< 0.01
Zone 3	1.47 ± 0.05	**2.66** ± **0.26**	**0.93**	0.06	< 0.01
Zone 4	1.87 ± 0.05	**2.03** ± **0.22**	**0.99**	< 0.01	< 0.01

*TP*  truncated power law, *P*  power law, *E*  exponential distribution

Table summarizes exponent *α* values and the maximum likelihood estimates, MLEs^70^, of the truncated power law exponents *μ* for cancer cell movements in vivo for the data described in Fig. 3 and 4 (± values give the 95% confidence intervals). Lower cutoff value was *a* = 5 min for all in vivo data sets. Away from the tumor, in zones 2–4, Akaike weights heavily favor truncated power law with *2* *<* *μ* *<* *3* (shown in bold) indicating L*é*vy walks for B16-F10. For B16F0 for all zones, distributions correspond to truncated power law with *μ* *>* *3* indicating diffusive walks. See Supplementary Movies 7 and 8. Statistics were based on the following numbers of cells and time points − B16-F0, zone #1 (*n* = 59 cells, *m* = 2,925 data poi*n*ts); zone #2 (*n* = 57, *m* = 2,806); zone #3 (*n* = 28, *m* = 1,383). B16-F10, zone #1 (*n* = 30 cells, *m* = 990 data points); zone #2 (*n* = 30, *m* = 990); zone #3 (*n* = 29, *m* = 957); zone #4 (*n* = 23, *m* = 759)

Most importantly, the cumulative frequency distributions, CFDs, of persistence times shown in Fig. 4d reveal that non-metastatic B16-F0 cells move diffusively (truncated power law with *μ* *>* *3*), whereas the metastatic B16-F10 cells have a CFD characteristic of a Lévy walk (truncated power law with 2 < *μ* *<* *3*). For the B16-F0 cells, the Akaike weights quantifying the appropriateness of the fits show that even though truncated power law fit is preferred over pure power law or exponential fits, *μ* *>* *3* indicates diffusive motion for all zones. For B16-F10 cells, truncated power law fit is also appropriate in all zones with Akaike weights close to 1, but the value of exponent 2 < *μ* *<* *3* for zones 2–4 indicates Lévy-like motion (Table 2). When analyzing all data for B16-F10 from zones 2–4 together, *µ* *~* 2.36 ± 0.13 with Akaike weights for truncated power law close to 1. Overall, these in vivo studies resemble the results obtained for cell’s on microtracks both in terms of the diffusive-vs-Lévy dichotomy and the linearity of cell trajectories (here, along tissue micro-channels rather than microtracks). We note that additional analysis of cells migrating through 3D collagen gels (to probe the effects of 1D vs. 3D microenvironment) are also consistent with diffusive-vs-Lévy motions for normal versus cancer cells (see Supplementary Note 4 and Supplementary Figures 8, 9).

### “Reprogramming” Lévy walks into other types of motions

Assuming that the Lévy-type walks might be an untoward characteristic of “predatory” metastatic cells, we next focused on the question whether these walks could somehow be altered—in particular—could they be reverted into diffusive motions characterizing the non-invasive cancer cells?

**Fig. 5:**
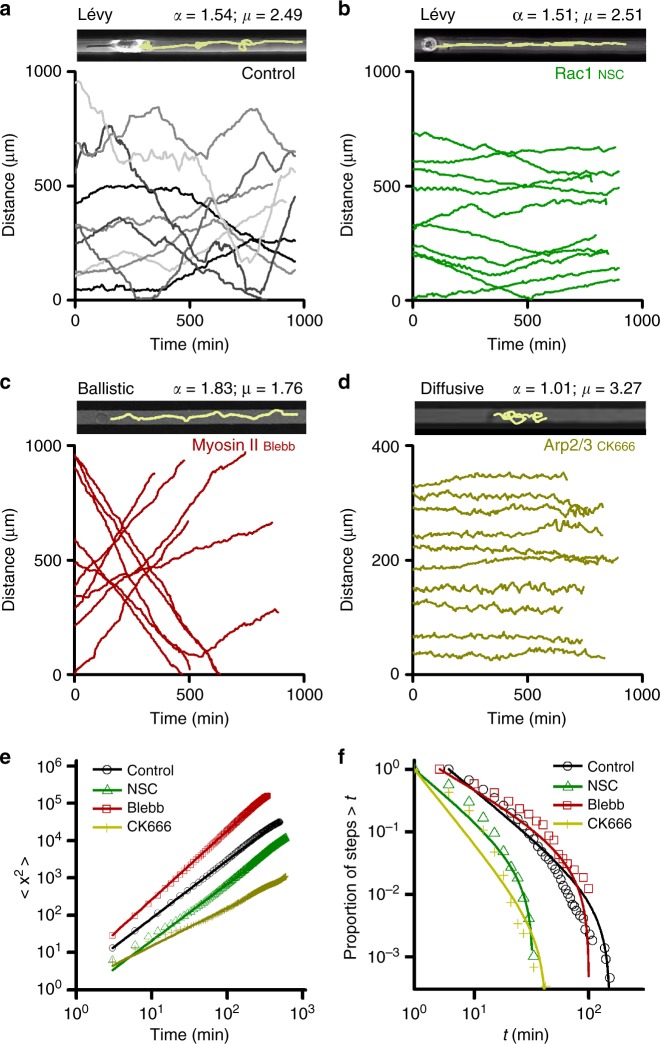
Altering the motility strategy of metastatic cancer cells. Examples of typical cell trajectories: Lévy walking (control MDA-MB-231 cells, **a** or with Rac1 inhibited, **b**), ballistic (Myosin II inhibited, **c**) diffusive (Arp2/3 inhibited, **d**). Line width = 20 μm, same for all three images. In displacement plots, ten representative trajectories for each treatment are shown. Quantification of motility characteristics (exponents μ, α along with the ± 95% confidence intervals) for MDA-MD-231 cells moving on microtracks and having individual actin-binding proteins inhibited is summarized in Table 3. **e** Log–log plots of the cells’ mean square displacement versus time, $\left\langle {x^2} \right\rangle = t^\alpha$. The slopes correspond to exponents α; note that inhibition of Arp2/3 with CK666 results in diffusive motion, α *~* 1, while inhibition of Myosin II results in ballistic motion, α*~*2. **f** The corresponding cumulative frequency distributions, CFDs, of persistence times. Markers are experimental statistics for persistence times. Solid lines are truncated power law fits with respective *μ* values shown in Table 3. For the chemical inhibitors data shown corresponds to: 40 μM CK666 (for Arp2/3, yellow crosses), 100 μM NSC23755 (Rac1, green triangles), 10 μM Blebbistatin (Myosin II, red rectangles), and control (black circles). The additional results for all drug and siRNA concentrations tested are shown in Supplementary Figures 10–13. See Supplementary Movies 9–12 and 16–18

To this end, we inhibited—either chemically or by using specific siRNAs—selected proteins known to be involved in polymerization of actin filaments and their organization into higher-order structures and known to drive extension of cellular protrusions^62^. Results summarized in Fig. 5 and Table 3 (see also Supplementary Movies 9–12 and 16–18**)** for the metastatic MDA-MB-231 cells moving on the microtracks reveal that inhibiting Rac1 (with chemical inhibitor NSC23766) or knocking down of Cofilin-1 (involved in actin filament depolymerization), Profilin-1 (regulating actin polymerization), or Dia-1 (involved in polymerization of unbranched actin filaments) increased *μ* exponents slightly, but not as much as to fully eliminate Lévy-like motions, (Table 3, see also Supplementary Figures 10–13). In contrast, inhibition of the Arp2/3 (but not of upstream GTPase Rac1, Fig. 5b and Supplementary Movie 11)— involved in the polymerization of actin into dendritic/branched networks at cell’s “front”—by a small-molecule inhibitor CK666 not only slowed the metastatic cells down, from 0.99 μm/min to 0.57 μm/min, but changed the nature of their motions from Lévy-like to diffusive (Fig. 5d, Supplementary Figure 14, Supplementary Movies 9, 17, and Supplementary Table 4 for the summary of all speed data). When Myosin II—typically involved in forming contractile actin networks and bundles and causing concurrent cell rear retraction^62^—was inhibited by blebbistatin at intermediate inhibitor concentrations (see  Fig. 5 legend), the cell speed changed only slightly from 0.99 μm/min to 1.13 μm/min, but caused cells to move ballistically (i.e., steadily in one direction; note that in the context of this work, “ballistic” corresponds to motions for which persistence times are longer than total observation time and for which α *~*2; Fig. 5c and Supplementary Movie 10). In addition, pronounced changes in the motility patterns could also be achieved by inhibition of several proteins simultaneously. A case in point here is the reversal of Lévy to diffusive walks by the simultaneous inhibition of Myosin II with 10 μM of blebbistatin and of Rac-1 with 100 μM of NSC23766 (Supplementary Figures 11 and 13, Supplementary Movies 12 and 18)—what is interesting about this result is that the inhibition of each of the proteins separately (cf. Fig. 5b, c) does not lead to diffusive walking, implying a synergistic/cooperative action of the two proteins in controlling the Lévy-walk movement pattern.

**Table 3:** Statistical analysis of metastatic cancer cell movements upon inhibition of actin regulators

Protein	MSD (*α*)	MLE for *μ*	Akaike weights		
inhibited		Truncated power law	TP	P	E
Control	1.54 ± 0.01	2.49 ± 0.07	> 0.99	< 0.01	< 0.01
Cofilin1_siRNA_	1.56 ± 0.05	3.01 ± 0.14	> 0.99	< 0.01	< 0.01
Profilin1_siRNA_	1.28 ± 0.01	2.62 ± 0.08	> 0.99	< 0.01	< 0.01
Dia1_siRNA_	1.51 ± 0.09	2.57 ± 0.11	> 0.99	< 0.01	< 0.01
Arp2/3_CK666_	**1.01** ± **0.03**	**3.27** ± **0.09**	> 0.99	< 0.01	< 0.01
Rac1_NSC23766_	1.51 ± 0.03	2.51 ± 0.08	> 0.99	< 0.01	< 0.01
Myosin II _Blebbistatin_	1.83 ± 0.09	1.76 ± 0.07	> 0.99	< 0.01	< 0.01

Table summarizes α exponent values and the maximum likelihood estimates, MLEs^70^, of the truncated power exponents *μ* for all treatments (± values give the 95% confidence intervals) described in Fig. 5. For the knockdowns, the values shown correspond to 30 nM siRNA concentrations. For the chemical inhibitors data shown corresponds to: 40 μM CK666 (for Arp2/3), 100 μM NSC23755 (Rac1), 10 μM Blebbistatin (Myosin II). The additional results for all drug and siRNA concentrations tested are shown in Supplementary Figures 10–14. Notice that Arp2/3 inhibition (highlighted in bold) reverts motions to diffusive characterized by *α* *~* 1 and truncated power law distribution with *μ* *>* *3*. Lower cutoff values were *a* *=* 6 for Control, Cofilin1, and Profilin1 siRNAs, and *a* *=* 3 for other treatments. Note that in all cases truncated power law (TP) distribution is favored over power law (P) and exponential (E) distributions, and diffusive-vs-Lévy characteristic is determined by the magnitude of the μ exponent. Analyses based on the following numbers of cells and time points: Control (*n* *=* 69 cells, *m* = 17,569 time points); Cofilin1_siRNA_ (*n* *=* 25 cells, *m* = 7,466 time points); Profilin1 _siRNA_ (*n* *=* 29 cells, *m* = 7,250 time points); Dia1 _siRNA_ (*n* *=* 7 cells, *m* = 2,017 time points); Arp2/3 _CK666_ (*n* *=* 36 cells, *m* = 9,008 time points); Rac1_NSC_ (*n* *=* 26 cells, *m* = 7,407 time points); Myosin II_Blebbistatin_ (*n* *=* 26 cells, *m* = 5,413 time points). See Supplementary Movies 9–12 and Supplementary Movies 16–18

### The role of protrusion-retraction synchronization in Lévy walks

Our previous morphodynamic profiling analyses have shown that in metastatic cells, the protrusions and retractions are highly “synchronized” both in space and in time; in contrast, protrusions and retractions formed by non-metastatic cells are not “synchronized”^63^. In order to test if such protrusion-retraction synchronization can give rise to truncated Lévy walk/power law step-size distributions, we developed a simple model described in detail in Supplementary Note 5, Supplementary Figures 15 and 16. Briefly, we considered a scenario in which the probabilities of taking left-right steps depend on prior history (non-Markovian process). We showed that if consecutive steps slightly favor moves in the “same” direction – e.g., as observed in the microscale dynamics of cell membrane where persistence of membrane protrusion typically depends on levels of actin regulators, such as Rac1 and Arp2/3, activation of the positive feedback loops, precise spatiotemporal regulation of Rho family GTPases, and coupling of protrusion to substrate adhesion^2,62^—then the overall distribution of persistence length can follow truncated power law (Supplementary Figures 15 and 16). The general conclusion of this modeling effort is, therefore, that synchronization (or desynchronization) of front-back protrusions/retractions may determine the overall motility pattern. The model indicates that cells in which front and back dynamics are synchronized are expected to perform truncated Lévy walks (as in our experiments with metastatic cells) whereas lack of synchronization should translate into diffusive motion (as in non-metastatic cells and metastatic cells treated with inhibitors). Experimentally, we observe some signatures of “desynchronization” of front-rear protrusion-retractions in MDA-MB-231 cells treated with inhibitors (or their combination) that revert Lévy to diffusive motion (see Supplementary Figure 14 for details and Supplementary Movies 16–18). Based on these results, we propose a hypothesis that Lévy-like movement pattern in metastatic cancer cells on 1D microtracks results from the balance/competition of persistent protrusion—via synchronized front-rear motions dependent on Arp2/3—and an effective myosin II-dependent mechanism for switching the direction of cell motion.

## Discussion

Taken together, the above results establish the presence of Lévy-like movement patterns in a range of metastatic cancer cells, and also suggest means of changing this motility phenotype. Metastatic cells not only move “faster and more persistently”^10^ than their non-metastatic counterparts, but while doing so, also display a fundamentally distinct path structure. This “predatory” navigation strategy is independent of external chemotactic signals as gradients of various chemokines, EGF, or VEGF are all absent in the microtrack experiments. This work contributes to the growing body of knowledge indicating that Lévy-like movement patterns are present and fundamentally important not only for humans^26,27^ and multicellular animals^17–25^, but also on cellular^6,28,29^ and subcellular levels^16^.

Lévy walk movement patterns are generally different from and should not be confused with classical models of cell motions such as persistent and simple correlated random walks (PRW/CRW reviewed by^1^). The latter are generally characterized by ballistic (i.e., persistent in direction) motions at short time and diffusive at long time scales. In contrast, Lévy walks are superdiffusive over long times thus allowing the searcher to move farther away from the starting point in a shorter time than CRW/PRW strategy would. This being said, it should be noted that recent theoretical analyses and experimental evidence from multicellular animal movements indicate that related movement patterns, specifically multi-phasic walks (e.g., composite CRWs and composite Brownian walks), in which the mover switches between two or more kinds of simple walk patterns (mathematically characterized by step-sizes from two or more exponential distributions which can sum up to a power law distribution^60^) have been suggested “to have parameters that are fine-tuned to [optimal] Lévy walk”^17,59^. While bi-phasic (or bi-modal) walks have realistic physical correlates (such as run phases and reorientation/wait phases)^64^, it seems difficult to find physical justification for fitting/using more complex multi-exponential distributions to describe step-size distributions, such as the ones we observe here for metastatic cells. In addition, discrete nature of cell motility data we collected precluded reliable fitting of bi-exponential distributions (see Supplementary Note 2 for more information).

While our observations of Lévy walk motility pattern for metastatic cells moving through very simple experimental environments in the absence of chemotactic gradients suggest that Lévy walk is an intrinsic property of metastatic cancer cells, current work does not exclude the possibility that constrained complex geometries, such as aligned collagen fibers and linear micro-tunnels/tracks, contribute to the emergence of the Lévy walk pattern in complex in vivo environments. In this context, it is interesting that recent theoretical studies have shown that linear constraints, such as one-dimensional micro-channels, provide one of the simplest systems for the emergence of optimal Lévy walks^23,32^. For example, in the system described by Reynolds et al.^23^, a Lévy-like displacement patterns (so-called Weierstrassian Lévy walk^23,29^) can emerge as a result of the moving object bouncing chaotically off the walls of the channel. While intriguing, such minimalistic mechanical explanation might not be at work in our 1D microtrack system simply because there are no “walls”, and cellular motions are determined mostly by cell–substrate adhesive interactions and are gently guided by adhesive micropatterns. At the same time, cellular interactions with complex geometric/mechanical constraints could play role in wider micro-channels/tracks, such as in PDMS micro-channels used by others^65^ and in vivo settings^36,43^.

To the best of our knowledge, the work presented here is the first demonstration of Lévy-like movement patterns in adherent mammalian cells, as well as in the context of metastatic cancer. Regarding the latter aspect, we ponder whether adopting the Lévy-like modality of migration might endow metastatic cells with a successful strategy for dispersing and searching for suitable loci where to seed new metastases. The prevailing theory (though disputed by others^,34^) stipulates that Lévy walk searches are considered to be optimal within a narrow range of circumstances^30–33^. Specifically, non-destructive Lévy walk searches with *μ~ 2* were considered optimal provided that targets are randomly distributed and scarce and searcher does not have prior knowledge about the target locations^30–32^. However, more recent theoretical work has shown that Lévy walk searches (again, with *μ~* *2*) are optimal under much broader environmental conditions than previously thought and that Lévy searchers experience fewer long periods of starvation (compared with exponential, composite Brownian and ballistic searchers)^33,66^. Furthermore, the so-called adaptive Lévy searching (where searcher has some knowledge of the target distribution and, upon target detection, responds by switching from extensive Lévy searching to intensive Brownian searching) has been shown to outperform adaptive ballistic and composite Brownian searches^67^. Interestingly, the theoretical 2D path structure analysis^33,34^ highlights a remarkable difference in movement patterns between Lévy (with *μ ~* 2.5, close to values observed in some of our experiments) and exponential searchers. The former takes many small steps in a focused area resulting in thorough focused exploration (high oversampling) until one large step takes it to another area (similar to 1D path structure of our metastatic cells, see Supplementary Movies 13–15). In contrast, an exponential searcher diffuses more gradually and covers the area more evenly without a thorough exploration of any particular spots^33^. This otherwise sub-optimal Lévy searcher (*μ~* 2.5) performs extremely well at intermediate levels of prey/target abundance. In the context of metastasis, focused local exploration characteristic of Lévy walk (with *μ~* 2.5) may be useful for finding “hot spots” of defective basement membranes^68^ followed by ballistic motions along linear collagen fibers and/or micro-channels toward another set of basement membranes before entering the bloodstream. Interestingly, risk of predation—condition relevant to metastatic cancer cells which during dissemination in vivo are constantly targeted by immune cells—alters optimal search strategy such that searchers executing Lévy walk with 2 < *μ* < 3 have higher fitness than those with *μ* ≤ 2^69^. Taken together, our work suggests that Lévy-like movements represent an emergent strategy of how highly motile metastatic cells move within complex microenvironments, and because such movement patterns could be advantageous, they may be maintained and/or fine-tuned by selection pressures.

Naturally, the present results should be generalized to as many types of cells/cancers and conditions (for example, micro-channels) as possible, though especially the in vivo studies are currently limited by the inability to visualize tumors lying deeper into the animal and to obtain long-enough trajectories. In addition, we do not yet fully understand the origin of Lévy walks at the level of cytoskeletal dynamics. While we have developed a simple model (see Supplementary Note 5 and Supplementary Figures 15, 16) that could ascribe these walks by the synchronization of the cell’s “front” protrusion (driven by Arp2/3-mediated nucleation of actin filaments) and “back” retraction (enabled by actomyosin contraction), such assumptions need further scrutiny and experimental validation. We see further effort in this area justified by the hope that by eliminating the ability of metastatic cells to execute “predatory” Lévy walks through the human body, it might be possible to decrease the ability of these cells to seed metastatic cancers.

## Methods

### In vivo observation of cell trajectories

The movements of stable Histone2B/EGF or Histone2B/mCherry expressing non-metastatic B16-F0 and metastatic B16-F10 mouse melanoma cells were observed in live tumors established in mouse dermis by using the dorsal skin-fold chamber model^43^. Dorsal skin-fold chambers were transplanted on a skin flap of C57/B16 J mice (Charles River) and B16-F0 or B16-F10 Histone2B-EGFP/mCherry cells were implanted by injection of 5 × 10^4^–2 × 10^5^ cells into the dermis adjacent to the deep dermal vascular plexus. For visualization of blood vessels, AlexaFluor 750-labeled 70 kD dextran was injected intravenously (2 mg/mouse). A customized multiphoton microscope (TriMScope-II, LaVisionBioTec) setup was used which allowed for simultaneous second-harmonic generation (SHG) to reconstruct tissue interfaces, and fluorescence imaging to track blood vessels and the movements of B16-F0 and B16-F10 cells stably expressing Histone-2B/GFP in live mouse dermis. Time-lapse acquisition was done 3–9 days after tumor cell implantation for 1–3 h at 5 or 10 min intervals.

All animal experiments were approved by the ethical committee on animal experiments and performed in the Central Animal Laboratory of the Radboud University, Nijmegen, in accordance with the Dutch Animal Experimentation Act and the European FELASA protocol (www.felasa.eu/guidelines.php).

### Tracking cell movements on 1D microtrack substrates

Tracks for cell locomotion were microetched in gold-on-glass substrates using the so-called Wet Etching^40,48,49^ technique (see Fig. 1a) where unetched gold regions were protected against cell adhesion with oligo(ethylene glycol)-terminated alkyl thiols, (HS(CH2)11(OCH2CH2)6OH (ProChimia Surfaces, Gdansk, Poland: www.prochimia.com). PC-3, PC3-M, MCF-7, MDA-MB-231, B16-F0 and B16-F1 cells were cultured according to the American Type Culture Collection (ATCC) protocol or as described previously^40,48,49^. Linear tracks were coated with Laminin (Sigma-Aldrich, cat. #L2020) or Laminin 5 (LN 5; extracted from 804G cells in crude form as previously described^52^). Cell migration was monitored on inverted microscope (TMD, Nikon) equipped with phase-contrast optics (10 × , 0.25 NA objectives) and CCD camera (Sensys, Photometrics, Tucson, AZ). Image acquisition was driven by Metamorph software (Universal Imaging Corp., Worchester, PA). Time-lapse videos were collected over 16 h in 3-min intervals. Cell positions were assigned by their center-of-mass coordinates.

### Data analysis

*Heavy-tailed models and fitting procedures:* The equations used in the comparisons of the heavy-tailed models can be found in Supplementary Table 1. Power law and exponential distribution parameters were estimated using analytical expression for maximum likelihood estimator, as discussed elsewhere^56,57^. Parameters for truncated power law, log-normal, and stretched exponential distributions were found using numerical maximum likelihood estimation and verified by visual inspection of likelihood maps. The appearance of largely negative *μ* values in case of log-normal model is demonstrated in Supplementary Figure 6. Effectively, the log-normal curve is being stretched to better fit power law-like distribution of the data, suggesting that log-normal is not the optimal model for the data. When using only positive *μ*, the resulting log-normal likelihood and, therefore, wAIC are worse. The resulting parameters are shown in the Supplementary Table 2. Parameter *a* is a lower cutoff parameter, the choice of which is described below, and *b* is an upper cutoff parameter used in truncated power law estimation, and corresponds to the maximal point in each dataset.

*Lower cutoff estimation:* To estimate the choice of the lower cutoff parameter *a*, we used a technique based on reweighted Kolmogorov–Smirnov (rKS) statistic, described in detail in ref. ^56^. In brief, the rKS is calculated for all *a* values taken from the set of unique values of persistence times in each dataset. Then, *a* is chosen where rKS is minimal and the number of remaining data points after thresholding by *a* is not less than 50% of the original dataset. In cases of truncated and regular power law, exponent values in Table 1 are shown for lower cutoffs determined separately using rKS, whereas *w*AIC for all distributions are calculated using cutoff values for the truncated power law.

For all other experimental details, please see Supplementary Methods/ Supplementary Notes 1–3 online.

## Electronic supplementary material

Supplementary Information

Description of Additional Supplementary Files

Supplementary Movie 1

Supplementary Movie 2

Supplementary Movie 3

Supplementary Movie 4

Supplementary Movie 5

Supplementary Movie 6

Supplementary Movie 7

Supplementary Movie 8

Supplementary Movie 9

Supplementary Movie 10

Supplementary Movie 11

Supplementary Movie 12

Supplementary Movie 13

Supplementary Movie 14

Supplementary Movie 15

Supplementary Movie 16

Supplementary Movie 17

Supplementary Movie 18

## Data Availability

Data and code used to generate results in the current study are available from the corresponding authors upon reasonable request.
