# Supplementary material for: Lévy-like movement patterns of metastatic cancer cells revealed in microfabricated systems and implicated in vivo
Source: Nat Commun. 2018 Oct 31;9:4539. doi: 10.1038/s41467-018-06563-w (PMC6208440; doi:10.1038/s41467-018-06563-w)
Supplement: Supplementary file 1 — Supplementary Information [file 41467_2018_6563_MOESM1_ESM.pdf]

Supplementary information

Lévy-like movement patterns of metastatic cancer cells revealed in microfabricated systems and implicated *in vivo*

Huda et al.

Table of Contents

Supplementary Figures…………………………………………………………………....3

Supplementary Methods and Discussion

Supplementary Note 1. Supplementary Methods………………………………………………..28

Supplementary Note 2. Additional considerations on the fitting bi-exponential distributions..37

Supplementary Note 3. Goodness-of-fit tests…………………………………………………....40

Supplementary Note 4. Analysis of cell trajectories in 3D collagen matrices……………..….42

Supplementary Note 5. A simple model of Lévy motion resulting from the synchronization of front/back protrusions/retractions…………………………………………………..….44

Supplementary Tables…………………………………………………………………...56

Supplementary References…………………………………………………………………60

Supplementary Figures

Supplementary Figure 1

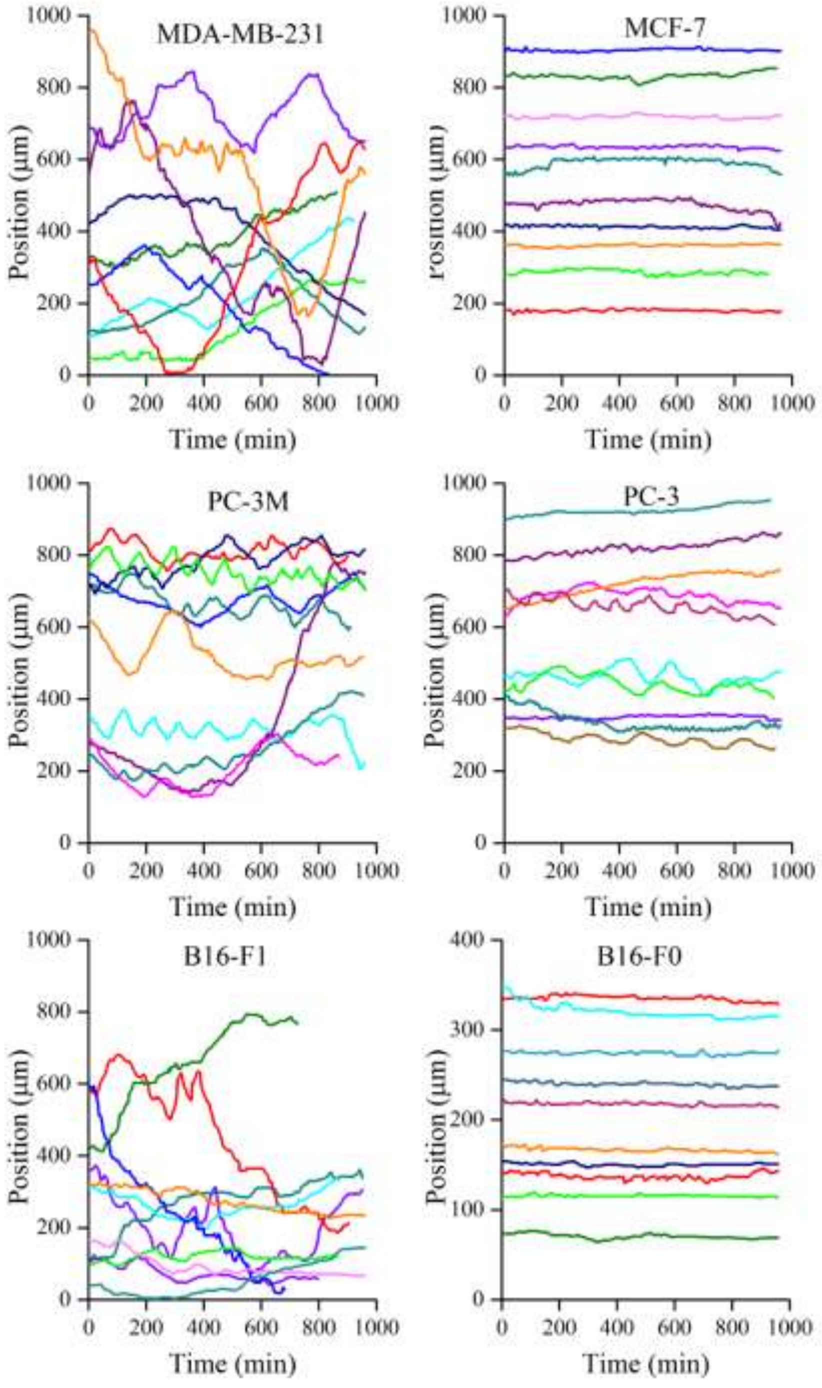

Supplementary Figure 1. Trajectories of cancer cell motions on linear microtracks. Typical trajectories/displacement vs. time (corresponding to analysis in Figure 2 and Table 1 in the main text) of highly metastatic MDA-MB-231, PC-3M, and B16-F1 cells feature characteristic small steps interspersed with unidirectional, long excursions. In contrast, trajectories of non-metastatic MCF-7, PC-3, and B16-F0 cells are more random/”jiggly”. Ten representative trajectories per cell type are shown. The starting points for trajectories are randomly positioned along the y axis (“Position”) for clarity. See also Supplementary Movies 1 – 6.

Supplementary Figure 2

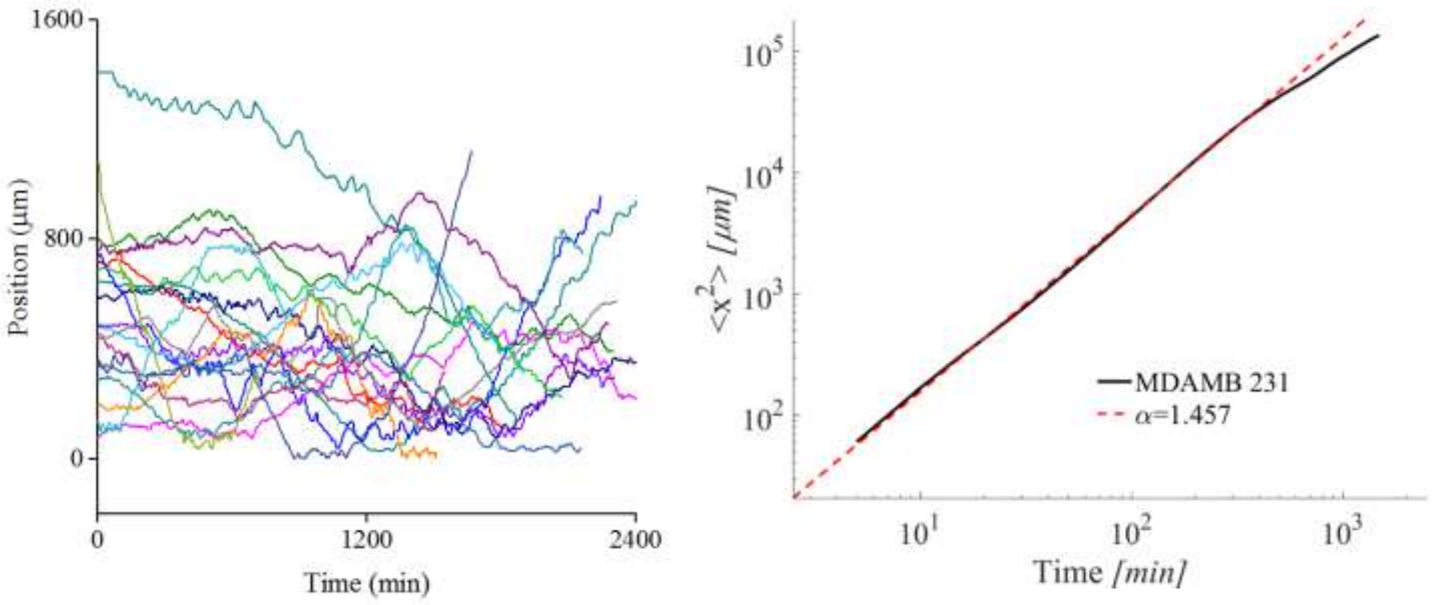

Supplementary Figure 2. Long-term trajectories of cancer cell motions on linear microtracks. Typical trajectories/displacement vs. time (corresponding to analysis in Supplementary Figure 5 and Supplementary Table 3) of highly metastatic MDA-MB-231 observed over 40h time period feature characteristic small steps interspersed with unidirectional,

long excursions. Eighteen trajectories randomly chosen from total of 49 cells are shown. See Supplementary Movies 13-15.

Supplementary Figure 3

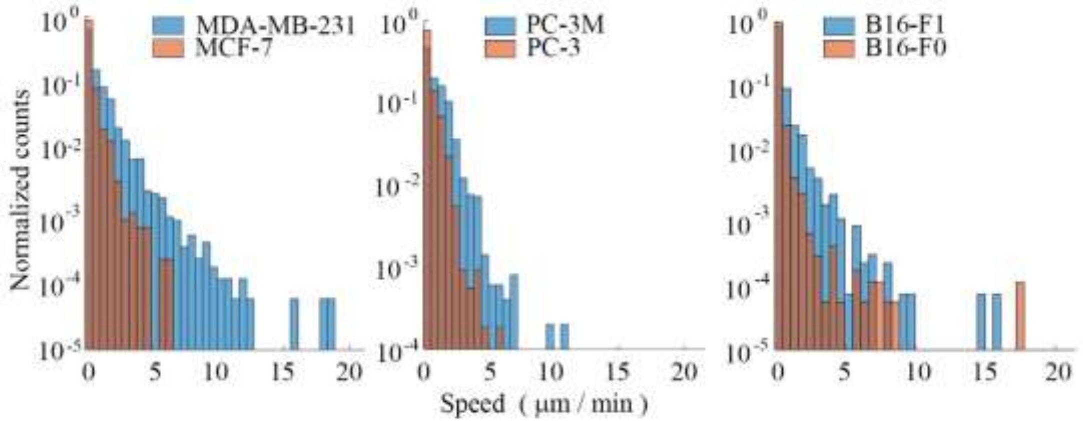

Supplementary Figure 3. Cell speed distributions corresponding to trajectories/analyses shown in Figures 1 and 2 and Supplementary Figure 1.

Supplementary Figure 4

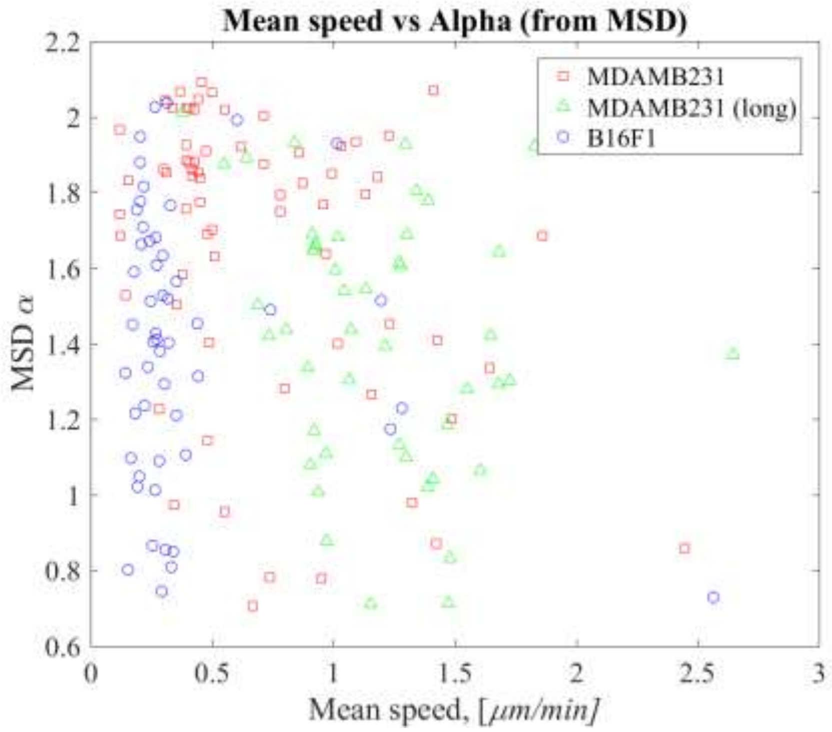

Supplementary Figure 4. Single cell motility parameters. We make three further observations about metastatic cell motions on single cell level: 1) Visual inspection revealed that even though individual trajectories appeared heterogeneous each trajectory featured "clusters of small steps" interspersed with long steps characteristic of Lévy walks (see Supplementary Figures 1 and 2 and Supplementary Movies 13-15); 2) Single cell mean speeds ranged from ~ 0.25 – 2 μm/min and $0.75 < \alpha < 2.1$ (see Supplementary Figures 3 and 4) , but clearly-cut subpopulations of cells could not be distinguished and majority of single cells moved superdiffusively with $1 < \alpha < 2$, and 3) faster cells were not necessarily most superdiffusive.

Supplementary Figure 5

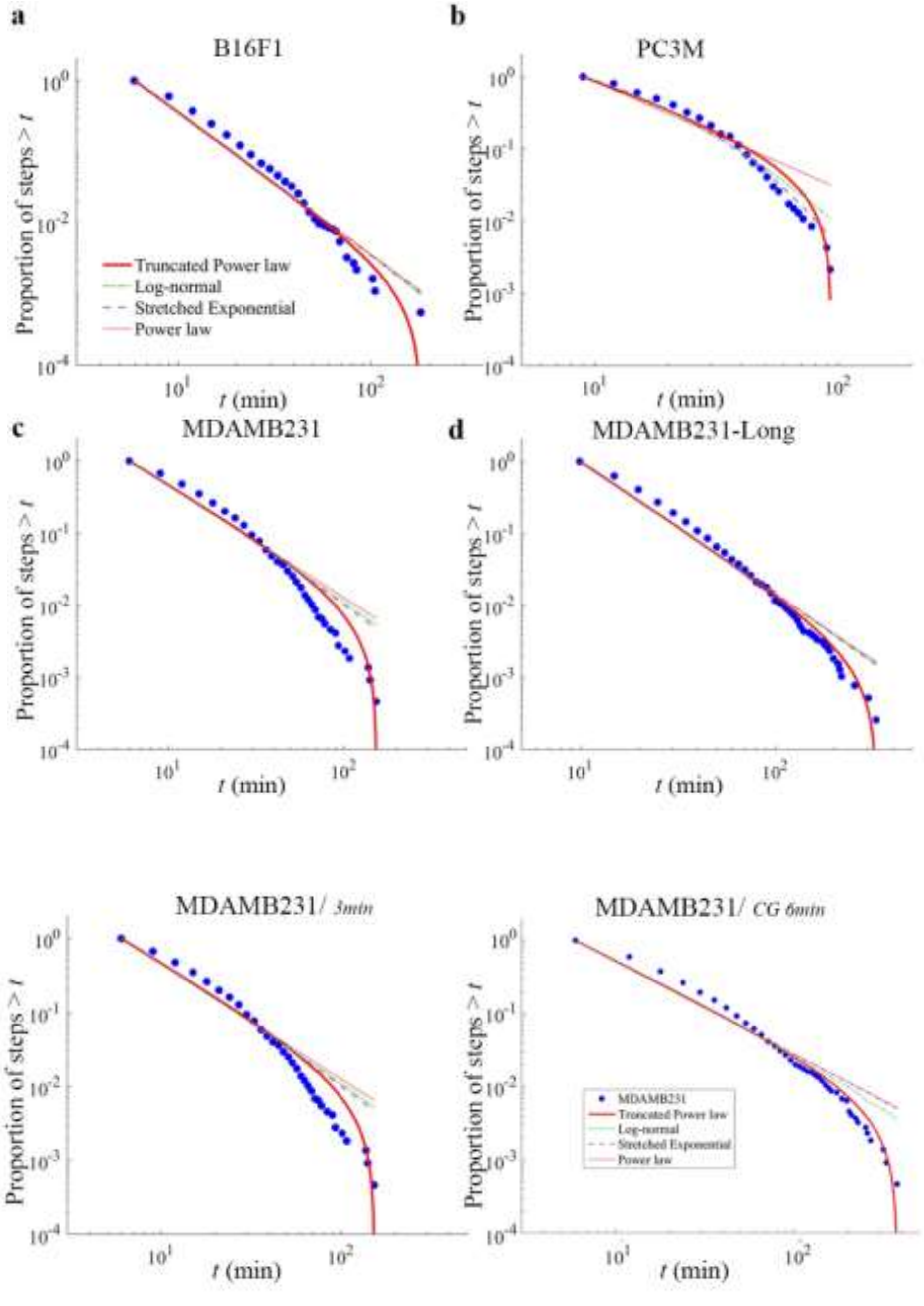

Supplementary Figure 5. Cumulative frequency distributions (CFDs) of persistence times and model fits for metastatic cells. (a-d) Plots correspond to data/analyses shown in Figure 2, Table 1 and Supplementary Figure 1 (on WETS substrates, 3 min time intervals) and MDA-MB-231 data set corresponds to long-term trajectory data shown in Supplementary Figure 2 and Supplementary Table 3 (long/CYTOO substrates, 5 min time intervals). Lower panel shows the same MDA-MB-231 data set analyzed without coarse graining of the data (*left*, 3 min time intervals) vs. with coarse-graining (*right*, 6 min time intervals obtained by discarding every second data point). Coarse-graining, in principle, could reduce the noise/tracking errors in trajectories arising from tracking the centroids of cells that continuously change their shape. While the CFD is shifted right reflecting capturing longer persistence time intervals, the overall conclusion – with truncated power law being the best fit – was the same when data was analyzed with (*right:* TP; μ =2.23) or without (*left:* TP; μ = 2.49) coarse-graining.

Supplementary Figure 6

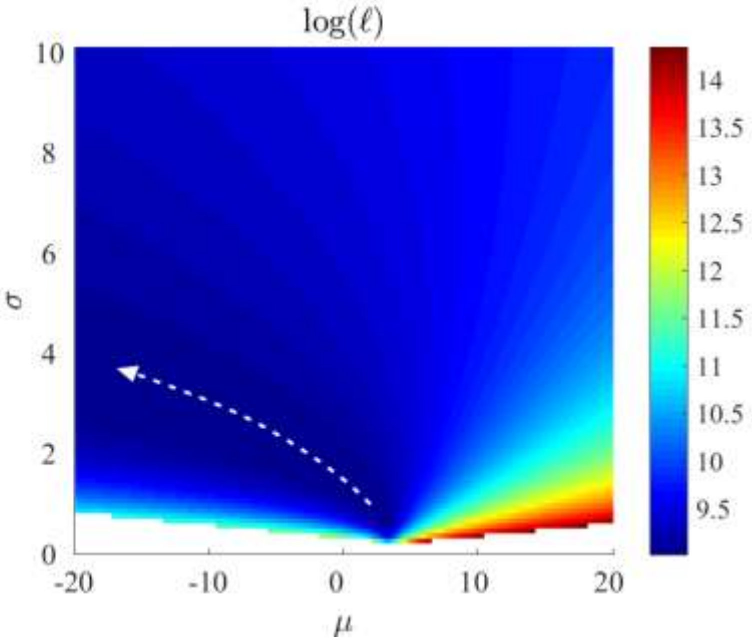

Supplementary Figure 6. Likelihood map of log-normal distribution for MDAMB231-long (CYTOO) dataset depending on the parameters μ and σ. Plotted in color is the logarithm of a negative log-likelihood, best fit parameters correspond to the minimum of the function. Negative log-likelihood decreases monotonically along the valley; the direction of decrease is shown by an arrow. Actual minimum lies far in the negative μ values beyond the scope of the picture.

Supplementary Figure 7

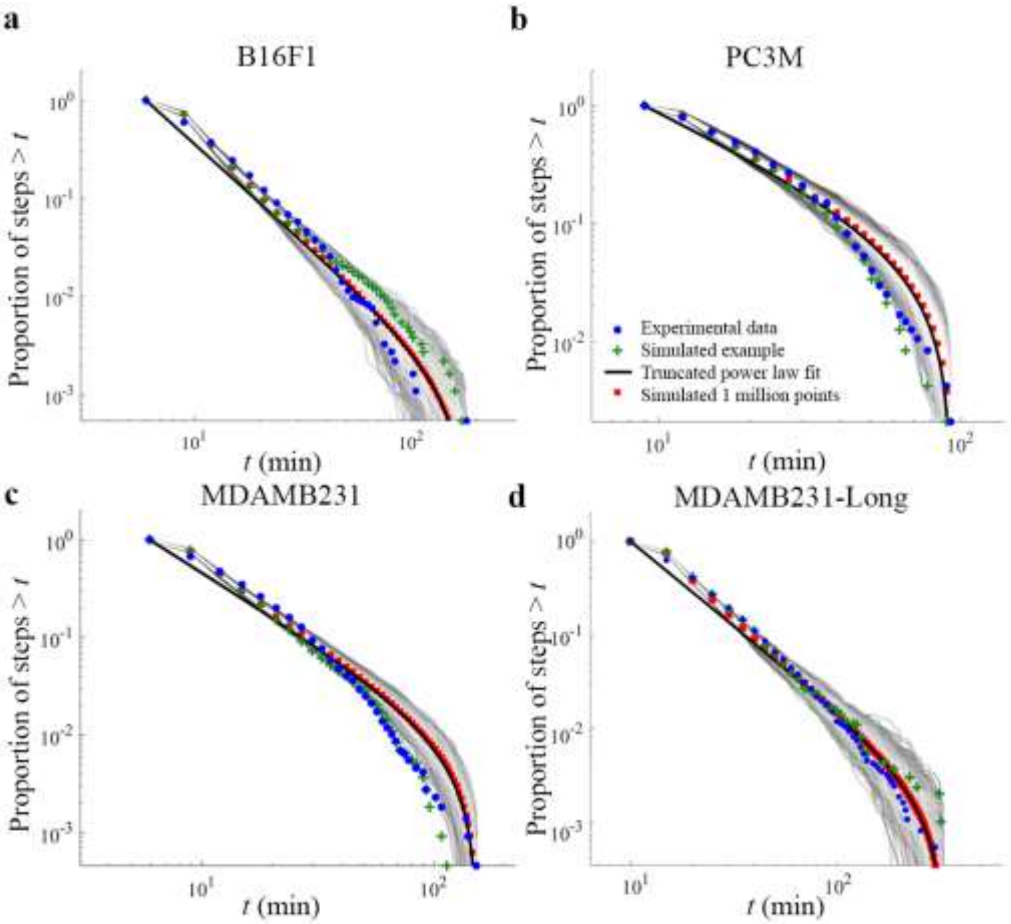

Supplementary Figure 7. Goodness-of-fit test simulations for metastatic cell data sets. Cumulative distribution functions (CDFs) for experimental data (*blue circles*), one random simulated data set example (*green crosses*) and truncated power law (TP) (*black curve*) with **(a)** μ = 2.99 for B16F1, **(b)** μ = 2.22 for PC3M, **(c)** μ = 2.49 for MDA-MB-231 and **(d)** μ = 2.8 corresponding to MDA-MB-231-long term data set. Discrete TP reference distribution (*red rectangles*) was generated by simulating one million data sets with interval between time points corresponding to experimental image acquisition frequency (frames per minute, fpm, were 5 min for MDA-MB-231-long data set and 3 min for all other data sets). Shaded curves in the background show one thousand of these simulated datasets shaded by KS distance away from the simulated TP reference (*red rectangles*) brighter color indicates data set being closer to the simulated TP reference distribution (*red rectangles*). The comparison of experimental data (*blue dots*) with simulated TP reference distribution (*red rectangles*) provides another way for graphically assessing the goodness-of-fit. Experimental data falling in the middle of the collection of the simulated data sets (shown in shades of grey) indicates that experimental data reasonably fits corresponding truncated power law model.

Supplementary Figure 8

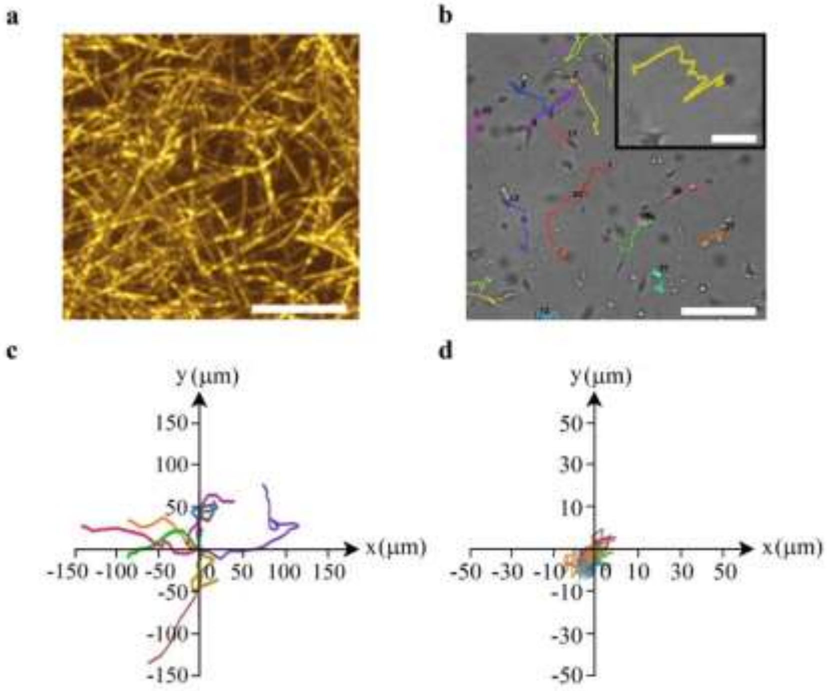

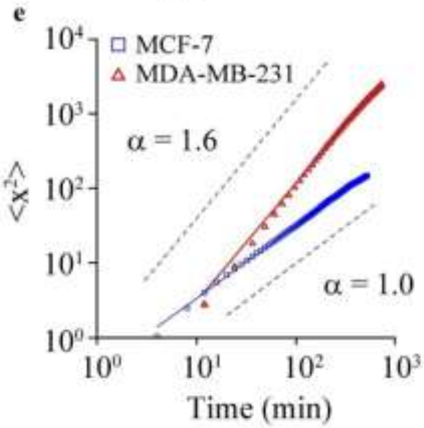

f

Cell type	α (95% CI)
Metastatic Cells	
MDAMB231	1.59 (1.57,1.61)
Non-metastatic cells	
MCF 7	0.97 (0.95,0.99)

Supplementary Figure 8. Superdiffusive migration and Lévy walks of metastatic cancer cells within collagen matrices. (a) Collagen migration substrate imaged by confocal reflection microscopy, Scale bar = 10 μm. **(b)** Cell movement in collagen gels (here, typical trajectories of metastatic MDA-MB-231 breast-cancer cells migrating for 16 hours; scale bar = 100 μm). Inset shows enlarged trajectory of one cell; scale bar = 30 μm. **(c)** Metastatic MDA-MB-231 and **(d)** non-metastatic MCF-7 trajectories over a 16 hour time period (here, *xy* slices are shown). **(e,f)** Plots, on a log-log scale, of the mean square displacement of trajectories versus time, $\langle x^2 \rangle \propto t^{\alpha}$. As in the microtrack studies in the main text, the trajectories in the collagen matrix are diffusive for MCF-7 cells (exponent α = 0.97), but superdiffusive for MDA-MB-231 cells (exponent α = 1.59).

Supplementary Figure 9

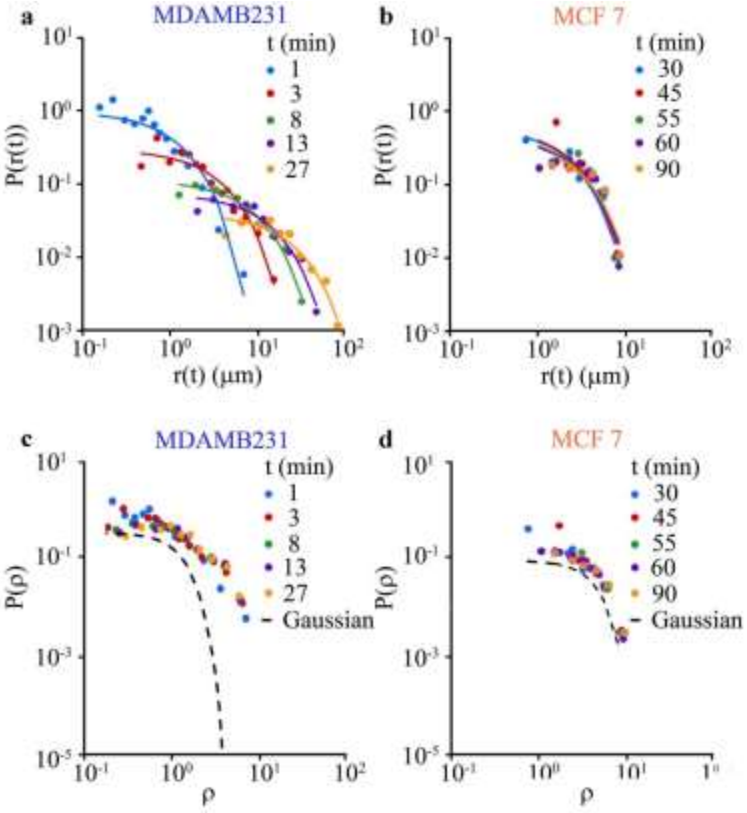

Supplementary Figure 9. **Lévy walks of metastatic cells in gel matrices. (a-b)** Time-sampled distributions of cell positions in collagen matrices. Legend gives the sampling times (longer for slower moving, non-metastatic cells). **(c-d)** When the distributions are appropriately rescaled (as described in Supplementary Note 4), all curves collapse onto one. For non-metastatic cells (such as MCF-7 shown here), the distribution fits a normal/Gaussian distribution, $p(r) = \frac{1}{\sqrt{2\pi\sigma^2}} e^{-\frac{r^2}{\sigma^2}}$ and indicates diffusive cell migration; for metastatic cells (here, MDA-MB-231), power-law dependence $P(r) = \left(\frac{\mu-1}{r_{\min}}\right)\left(\frac{r}{r_{\min}}\right)^{-\mu}$ is observed indicating Lévy walking.

Supplementary Figure 10

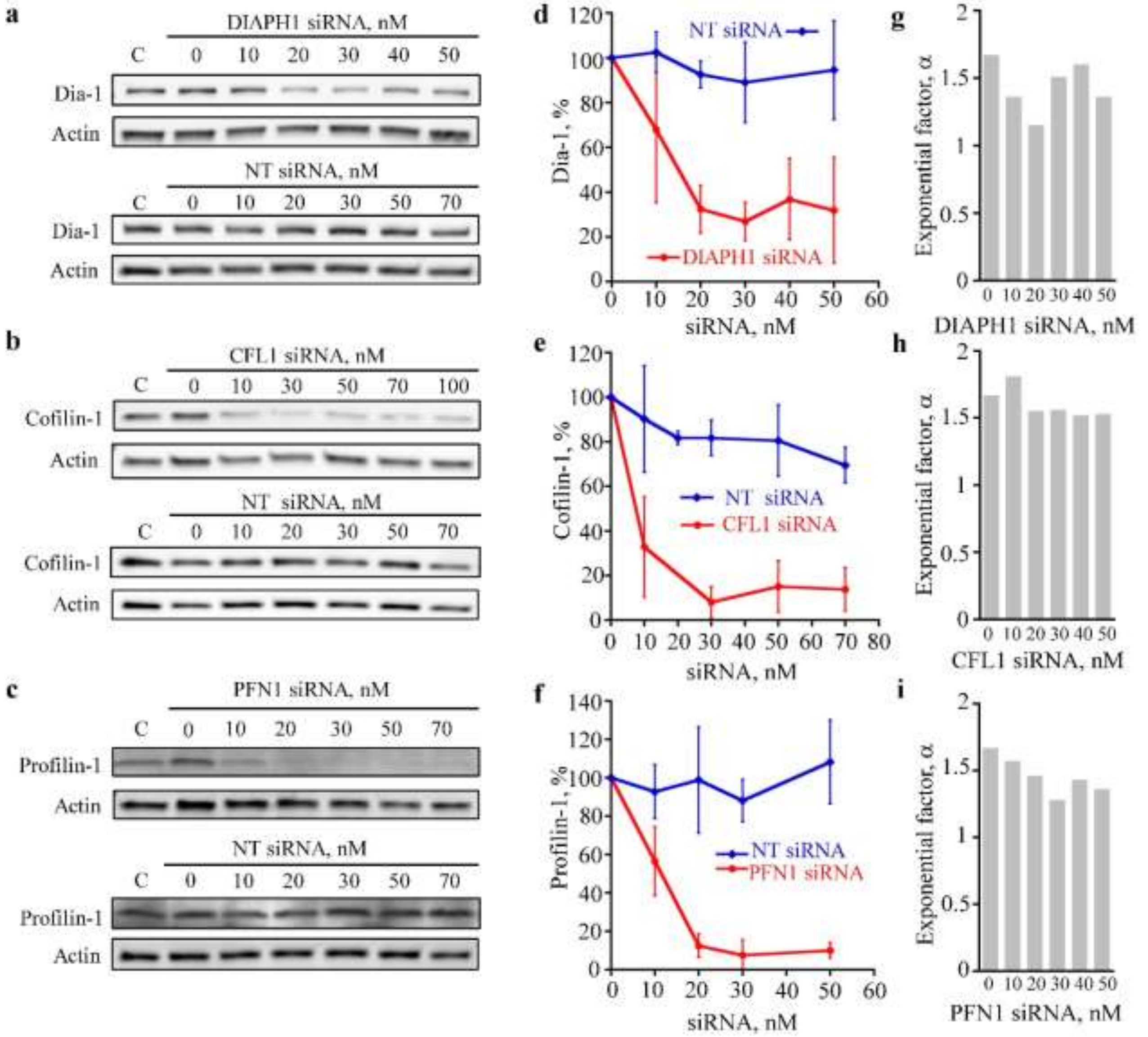

Supplementary Figure 10. The effect of depletion of actin regulators with RNA interference on cell motions. The depletion of actin regulatory proteins Dia-1 (encoded by DIAPH1 gene), Cofilin-1 (CFL1) and Profilin-1 (PFN1) by treatment with various concentrations (0 -70 nM) of corresponding siRNAs for 72 hours was validated with Western blotting. (**a-c**) Representative Western blots for cells treated with DIAPH1, CFL1, PFN1 or non-targeting (NT) siRNAs; C = untreated control. (**d-f**) Quantification of protein levels remaining after cell treatments with corresponding siRNAs. All targeting siRNAs induced good knockdowns (<25% endogenous protein remaining at 30 nM siRNA), while NT siRNA had negligible effect on target protein levels. **(g-i)** MDA-MB-231 cells treated with siRNAs were plated onto the linear microtracks and imaged for 16 hours at 3 minute intervals to yield α exponents.

Supplementary Figure 11

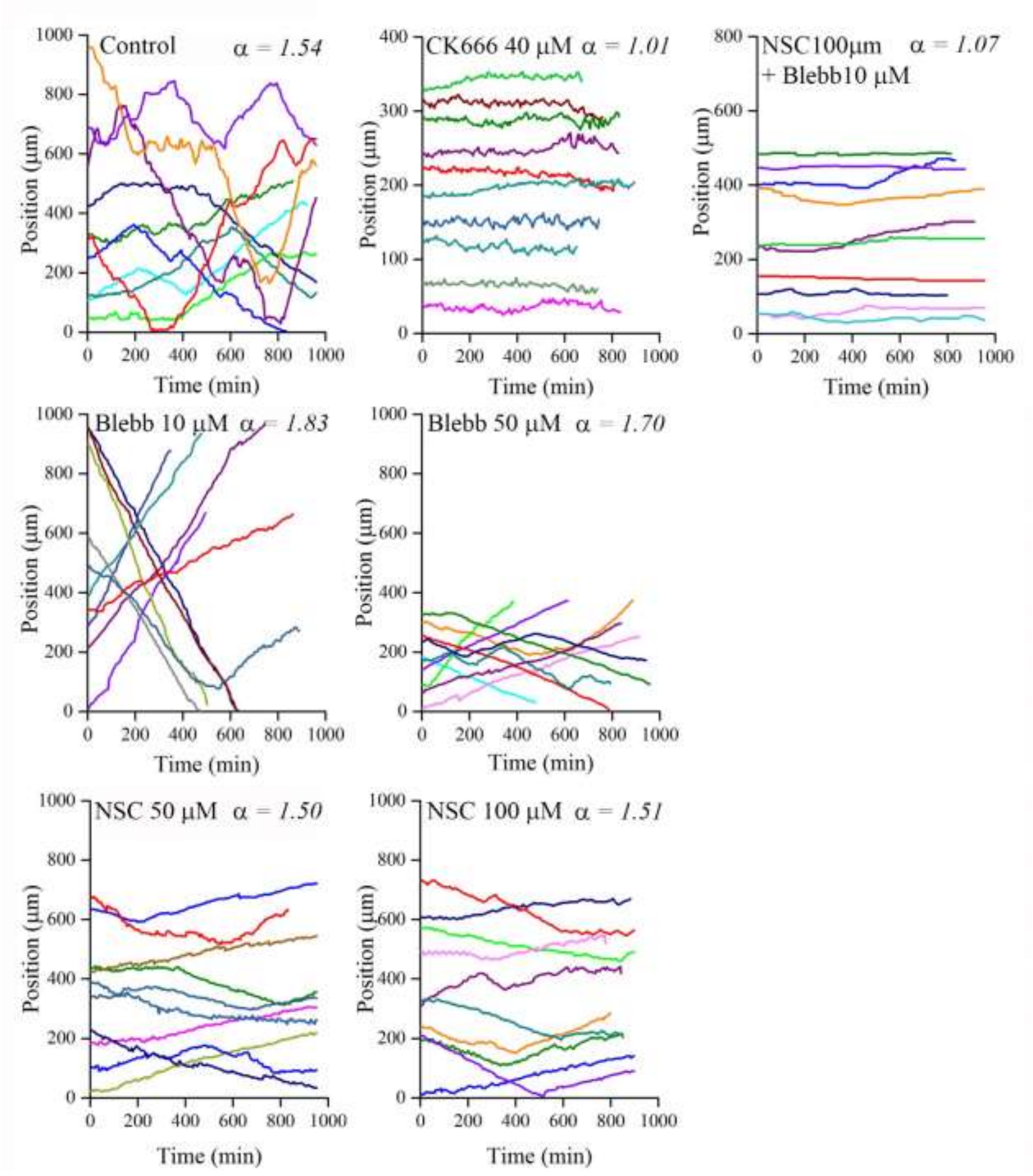

Supplementary Figure 11. **Representative cell trajectories observed during drug inhibition studies.** Ten representative trajectories are shown for each treatment group. Number of cells tracked were as follows: 69 cells for Control, 35 cells for CK666, 36 cells for NSC + Blebb, 26 cells for blebbistatin 10 μM, 17 cells for blebbistatin 50 μM, 29 cells for NSC 50 μM and 26 cells for NSC 100 μM. Partial inhibition of Myosin II with low concentration (10μM) of blebbistatin lead to ballistic motion with $\alpha = 1.83$. The treatment of cells with 50 μM of blebbistatin, which should be enough to completely inhibit Myosin II, lead to slower motion, with trajectories still appearing ballistic and $\alpha = 1.70$. Inhibition of Rac1 with either 50 or 100 μM of NSC23755 did not revert cellular motions to diffusive.

Supplementary Figure 12

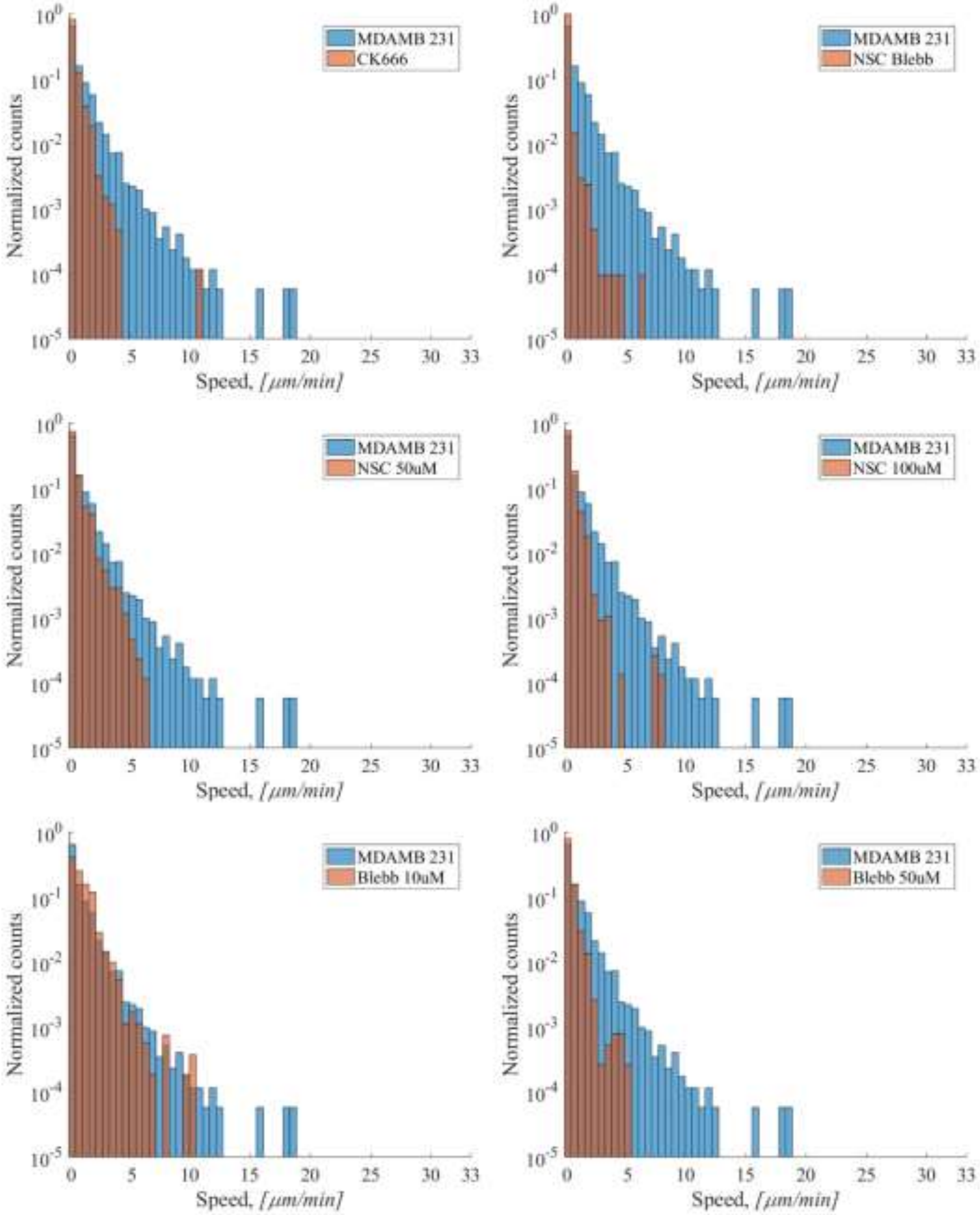

Supplementary Figure 12. Cell speed distributions for MDA-MB-231 cells treated with various inhibitors (corresponding to the trajectories and analyses shown in Figure 5 and Supplementary Figure 11).

Supplementary Figure 13

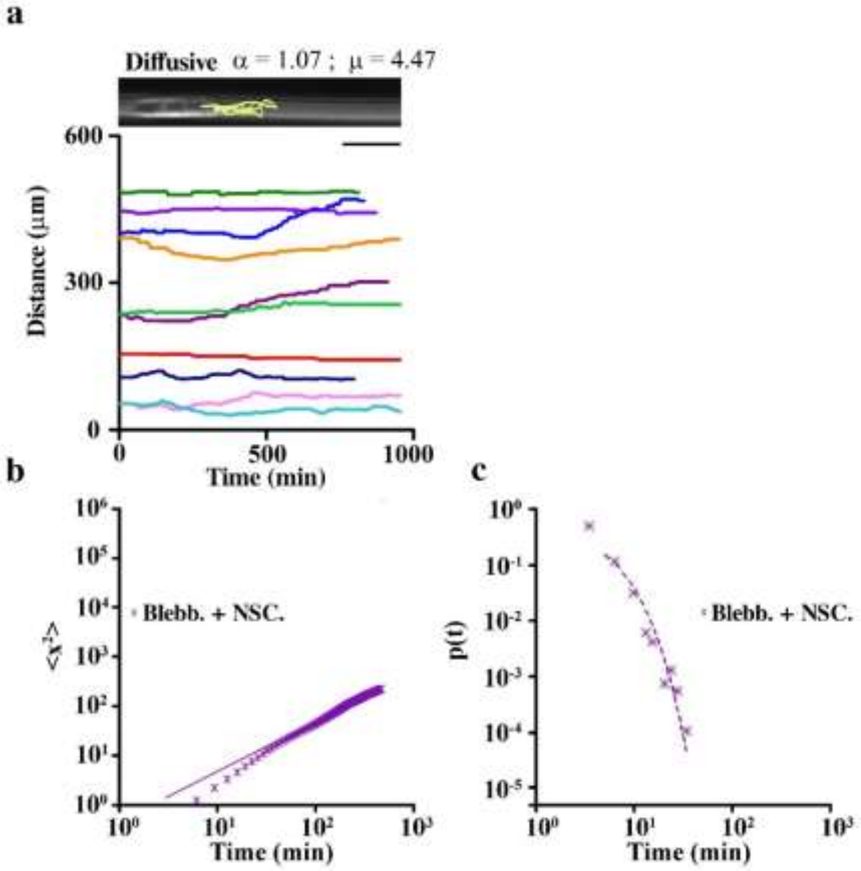

Supplementary Figure 13. Altering the motility strategy by double inhibition. Quantification of motility characteristics (exponents μ, α along with the ± 95% confidence intervals) for MDA-MD-231 cells moving on microtracks with simultaneous inhibition of Rac1 and Myosin II. Rac1 was inhibited by NSC23766 (100 μM) and Myosin II was inhibited by Blebbistatin (10 μM). **(a)** Ten representative trajectories. **(b)** Log-log plots of the cells' mean square displacement versus time, $\langle x^2 \rangle \propto t^\alpha$. **(c)** The corresponding probability distribution function. μ = 4.47 ± 0.21; α = 1.07 ± 0.01 ; Akaike weights: TP = 0.55; P = 0.44; E < 0.01 ; reflective of diffusively moving cells.

Supplementary Figure 14

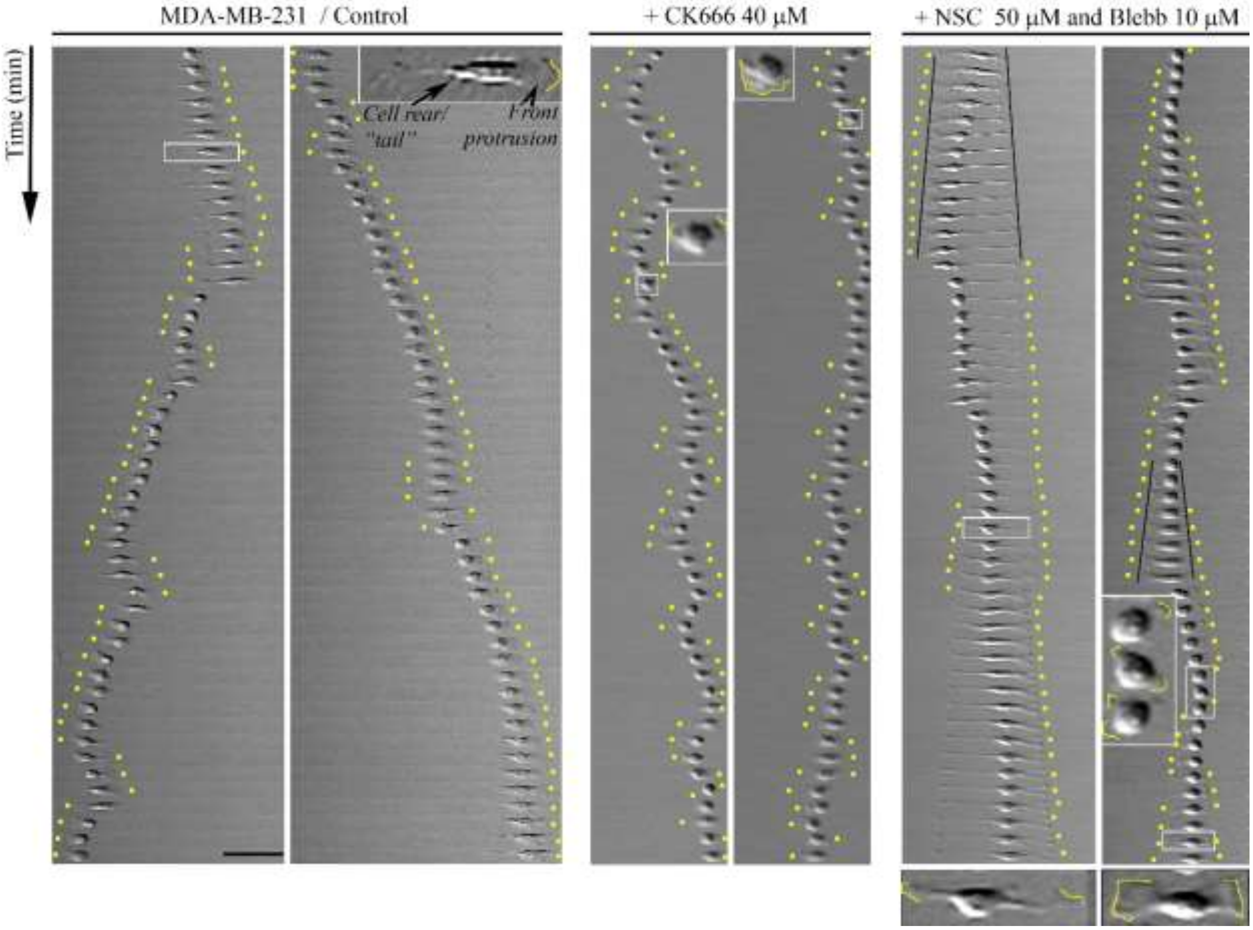

Supplementary Figure 14. The analysis of front-rear protrusion-retraction synchronization. The montage of successive frames (5 min per frame) from the time-lapse movies showing MDA-MB-231 cells moving along 20 μm-wide micropatterned lines. MDA-MB-231/Control cells represent Lévy walking cells, while CK666 or NCS+Blebbistatin treatment reverts cellular motions to diffusive (corresponding to analyses in Figure 5). Note that unlike in Figure 1, here linear micropatterns (CYTOO motility chip, 20 μm lines) are invisible. To illustrate the protrusion morphology/dynamics, two examples of non-consecutive 250-min intervals for each treatment are shown. Cells imaged with differential interference contrast mode (DIC) (see Methods section S2 in SI) feature easily discernable cell body (high-contrast regions) with less-well resolved (adjacent to cell body) thin membranous protrusions, so-called lamellipodia. For clarity, the direction of the protrusion extension is indicated on each line by yellow dot (or a dot on each side if cell is bi-polar) and in insets by yellow lines. Front-rear protrusion-retraction is considered "synchronized" when cell's front moves in concert with the cell's rear; conversely, "de-synchronization" refers to cell's front not moving in concert with the rear.

MDA-MB-231/ Control: Untreated MDA-MB-231 cells on linear microtracks displayed polarized, elongated mesenchymal morphology with clearly discernable front (front protrusion is indicated with yellow line in tear-drop shape shown in the inset) and rear. Both "clusters of short steps" (*left image*) and "long" steps (*right image*) characteristic of Lévy walks were identifiable. During "long" steps synchronized front-rear translocation along the microtracks was observed; occasional desynchronization events (visually corresponding to cell elongation signifying faster front protrusion than tail retraction or cell shortening indicating slower front protrusion than rear

retraction) were observed, but these did not always correspond to the change in direction. See Supplementary Movie 16.

MDA-MB-231 + CK666: Cells treated with Arp2/3 inhibitor CK666 displayed more compact cell shape featuring short (suggesting defective lamella) but polarized protrusion in most frames and lack of elongated "tails". When translocation of cell body was observed (*left image*), motions of front-rear appeared synchronized, but this "synchronization" did not persist. Protrusions frequently switched the direction between successive frames (*right image*) consistent with diffusive motions of these cells. From time to time, cells depolarized and extended small protrusions randomly around the circumference of the cell body *(see insets*) suggesting desynchronized front/back protrusions/retraction at these time-points. See Supplementary Movie 17.

MDA-MB-231 + NSC and Blebbistatin: Two types of morphological features were predominant—(*left image*) multiple non-productive stalk-like protrusions and (*right image*) non-productive (e.g., not resulting in translocation of cell body) bursts of several – at least two but also more – simultaneous lamellipodia protruding in different directions (best appreciated in the movie, see Supplementary Movie 18). Two types of features suggest desynchronization of front-rear movements – "bipolar" cells that extend lamellipodia in two directions (see inset), and instances where dominant lamellipodia (= cell front) moves forward while rear stays stationary, or at extreme extends in opposite direction (marked with black lines).

Scale bar is 100 μm.

Supplementary Figure 15

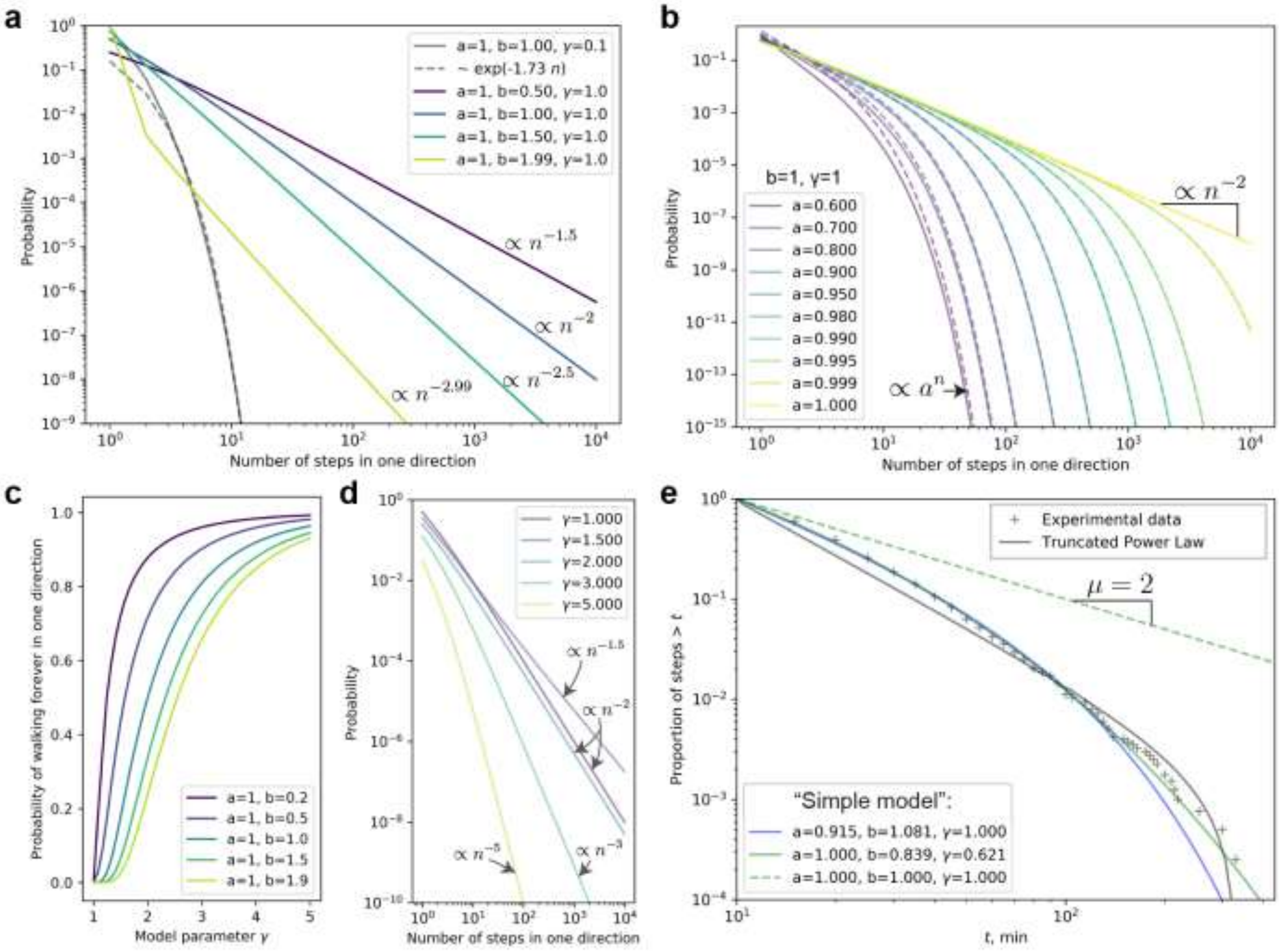

Supplementary Figure 15. Features of the random process generated by probability $q_k = a - b(1+k)^{-\gamma}$ of continuing motion in the same direction after k steps has been made in this direction**. (a)** Power-law behavior of probability distribution of persistence lengths for different values of parameter b and fixed $\{a = 1, \gamma = 1\}$. Also shown is approximately exponential decay of persistence lengths for $\{a = 1, b = 1, \gamma = 0.1\}$. **(b)** Distributions of persistence lengths for $\{a < 1,\ b = 1,\ \gamma = 1\}$. Different values $a < 1$ produce exponential cut-off at persistence lengths of order $(-1/\ln(a))$. **(c)** Probability of walking forever in one direction when $\{a = 1, \gamma > 1\}$. **(d)** Distributions of persistence lengths when $\{a = 1, b = 1, \gamma > 1\}$ for those cells that don't happen to walk forever in one direction. Asymptotic power laws are indicated. **(e)** Best fit of discrete model with fixed $\gamma = 1$ (blue curve) and all three parameters optimized (solid green curve) to experimental CFD for MDAMB231 long-term dataset. Best fit of Truncated Power Law obtained in Supplementary Figure 5 is shown as black curve for comparison. Length of step of the discrete model is chosen to be equal to the experimental time bin (5 min).

Supplementary Figure 16

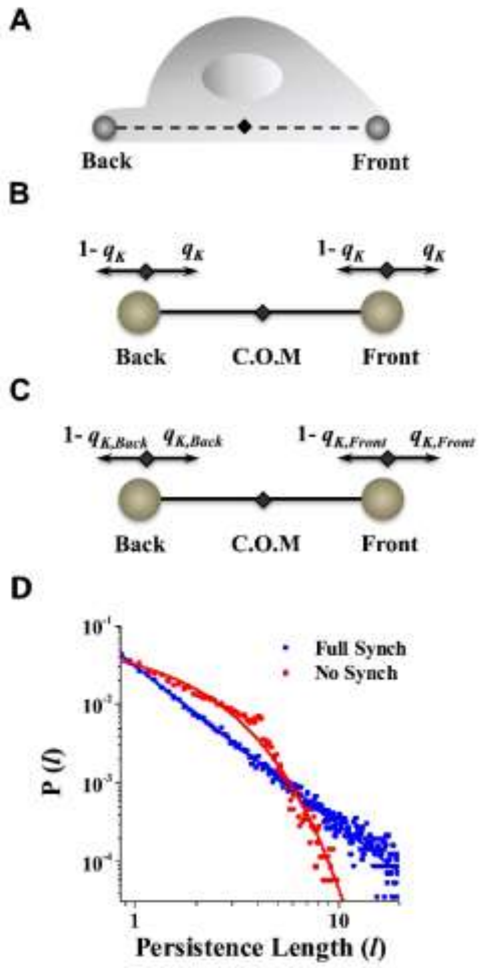

Supplementary Figure 16. Lévy motion can result from synchronization of front/back protrusions/retractions. (a) Illustration of a minimal model in which a motile cell (gray) is represented by connected "front" and "back" each having its own protrusion/retraction dynamics. Black diamond represents cell's center of mass. The probabilities of motion for connected front and back points for **(b)** fully synchronized motions, and for **(c)** motions without front-back synchronization. **(d)** Full front-back synchronization results in a power-law distribution of persistence lengths (blue markers) characteristic of Lévy walks. Without synchronization, the distribution is Gaussian (red markers) and indicative of diffusive motions.

Supplementary Note 1. Supplementary Methods

Microtrack substrate preparation

Tracks for cell locomotion were microetched in gold-on-glass substrates using the so-called Wet Etching [1-3] technique (see Figure 1a in the main text). Briefly, agarose stamps were prepared by casting hot, degassed agarose solution (10% w/w) against micropatterned, plasma-oxidized poly (dimethyl siloxane) (PDMS) masters. After gelation, the stamps were soaked in a gold etchant solution (Transene Co. Inc, Danvers, MA; 1:4 dilution in deionized water) for 15 min, air dried face-up for 1 min, equilibrated face-down on a glass slide for 1 min, and dried under a stream of nitrogen for 30 sec. For etching, stamps were placed face-down onto standard glass coverslips (thickness #1.5, area 22x22 mm^2) coated with an e-beam evaporated gold layer (30 nm) supported by a titanium adhesion layer (5 nm). Etched substrata were washed with ethanol (200 proof), and dried under nitrogen stream. Remaining gold regions were protected against cell adhesion by incubation in 5 mM solution of oligo (ethylene glycol)-terminated alkyl thiol ($HS(CH_2)_{11}(OCH_2CH_2)_6OH$; EG_6 (ProChimia Surfaces, Gdansk, Poland: www.prochimia.com) for 12 hrs. Protected substrates were thoroughly rinsed with ethanol, and dried under nitrogen stream.

Cell culture for the 1D microtrack studies

PC-3 (from ATCC, catalog# CRL-1435) and PC3-M (provided by Dr. Jill Pelling; Northwestern University, Evanston, IL, USA), MCF-7 and MDA-MB-231 (from ATCC, catalog # HTB-22 and HTB26, respectively), B16-F0 and B16-F1 cells (ATCC, catalog # CRL-6322 and CRL-6323, respectively) were cultured according to the American Type Culture Collection (ATCC) protocol or as described previously[1-3]. In particular, MDA-MB-231, MCF-7, B16-F1 and

B16-F0 were cultured using Dulbecco's modified Eagle's medium supplemented with 10% fetal bovine serum (FBS) at 37°C and 10% (for B16 cell lines) or 5% (all other cell lines) CO_2. PC-3 and PC-3M cells were cultured in Roswell Park Memorial Institute (RPMI) medium supplemented with 10% FBS at 37°C in 5% CO_2. Cell lines were not independently authenticated. Cell lines were tested for mycoplasma contamination and found to be mycoplasma free.

Linear tracks were coated with the desired extracellular matrix protein, Laminin (Sigma-Aldrich cat. # L2020) or Laminin 5 (LN 5; extracted from 804G cells in crude form as previously described,[4] and cells were plated onto them and allowed to spread for at least 2 hrs prior to imaging. MDA-MB-231, MCF-7, B16-F1 and B16-F0 cells were plated onto substrates coated with Laminin, while PC-3 and PC-3M cells were plated onto Laminin 5. In all experiments, cells were plated at a density of 10,000/cm^2 such that ~ 60% of tracks contained only one cell.

Cell culture in collagen matrices

Cells were cultured according to protocols detailed in Ref[5,6]. Briefly, cells were detached by EDTA (2mM) and incorporated into collagen matrices consisting of native dermal bovine type I collagen (Purecol, Nutacon) at 1.7 mg/mL and physiologic pH. Collagen lattices were overlaid by medium yielding a 6% final FBS concentration.

Time-lapse microscopy of cell migration

Cell migration was monitored on inverted microscopes (TMD, Nikon for microtracks and Leica, DMIL for collagen studies) equipped with phase-contrast optics (10X, 0.25 NA objectives) and CCD cameras (Sensys, Photometrics, Tucson, AZ). Cells in the collagen matrices were also monitored by confocal microscopy according to protocols detailed in Ref[5,6]. Image acquisition

was driven by Metamorph software (Universal Imaging Corp., Worchester, PA) for the microtracks, or time-controlled 16 channel recorder' Software (SVS-Vistek GmbH), for collagen migration. For both types of studies, observation started 1-2 hrs after cell plating or incorporation into collagen for all cell types, except for PC3-M and PC-3 cells that were allowed to spread and to polarize for 16 hrs before imaging. Briefly, before time-lapse observation of cell motions on microtracks, media was exchanged to Leibowitz-15 (L-15) supplemented with FBS – 2% for B16 cell lines and 10% for all other cell lines. To prevent evaporation and environmental contamination, the medium in the dish containing the microtracks was overlaid with mineral oil, whereas for collagen experiments, closed chamber systems containing equilibrated conditions were used. Time-lapse videos were collected over 16-20 hrs in 3-4 min intervals (note: time intervals of 20 and 10 minutes were also tested but were too long to capture all necessary characteristics of cell trajectories such as rapid changes in migration direction; shorter time intervals [e.g., 1 min] gave conclusions identical to those obtained at 3-4 min intervals). Cell positions were assigned by their center-of-mass coordinates.

A preliminary series of experiments was performed in which the width of the microtracks, w, was varied from 10 μm to 50 μm. On tracks thinner than ca. 15 μm, cells of all types tested moved significantly slower than on unconstrained, two-dimensional surfaces; on tracks 25 μm or wider, the cells moved not only along the track but also transverse to its long axis. Consequently, we used 20 μm wide tracks on which cell motions were, to a good approximation, one-dimensional while cell velocities were similar (to within ~5%) to those of the same cells on "unconstrained," two-dimensional substrates.

1D experiments were designed with standard experimental procedures for collecting single cell motility data in mind, e.g., trajectories from both microtrack and collagen migration analyses, in which cells “collided” with one another, were excluded from analysis. The sole reason for not including colliding cells was to, in principle, enable collection of data on “long excursions” (which could be shortened if cell collisions changed cells’ direction of motion). However, our visual observation was that colliding cells still were capable of performing long excursions, but trajectories of colliding cells were not analyzed rigorously.

For long-term experiments, (specifically MDA-MB-231 long-term data set #2 showed in Supplementary Movies 13-15 and analyzed Supplementary Figures 2 and 5 and Supplementary Table 3), cells were plated and cellular motion recorded on motility chips (CYTOO, Grenoble, France) featuring 20-μm wide micropatterned linear microtracks coated with Laminin. Cells were labeled with Cell Tracker Green CMFDA dye (4 μM; ThermoFisher Scientific cat# C7025), cell motions were monitored by confocal microscope (Nikon A1R) equipped with 10X objective (10X, 0.25 NA objective) over 40 hrs in 5 min intervals. Chamlide incubator system (Live Cell Instrument, Seoul, South Korea) was used for maintaining environmental conditions during observation. Cell motions were tracked based on fluorescence images by using Imaris software (Bitplane, Zurich, Switzerland). The cellular motion pattern and statistics were similar to those for cells moving on micro-etched gold-on-glass substrates and on CYTOO motility chips.

In vivo observation of cell trajectories

The movements of non-metastatic B16-F0 and metastatic B16-F10 mouse melanoma cells were observed in live tumors established in 8-14 week old male C57Bl/6 J (Charles River) mouse

dermis by using the dorsal skin-fold chamber model. B16F10 (ATCC; cat# CRL-6475) and B16F0 (ATCC; cat# CRL-6322) were from ATCC and were regularly tested negative for mycoplasma. The number of animals was based on the number of individual tumor cells and invasion zones which could be analyzed per mouse. Data was acquired from at least 3 independent experiments. We excluded tumors of all groups from the analysis which were too deeply injected into the subcutaneous fat tissue. The particular tissue structures of subcutaneous fat tissue changes the invasion pattern of tumor cells and results would not be comparable to the tumors growing in the deep dermis. Following the application of the imaging window, we excluded mice with inflamed imaging windows and randomly divided the mice into two groups (researcher A) receiving either B16F10 or B16F0 tumors (researcher B). The animals were housed individually and cages were numbered without identification of groups. Thus, for housing the biotechnician or researcher was blinded. During image acquisition and analysis the difference between the invasion pattern of B16F10 and B16F0 was quite obvious so that true blinding was impossible to achieve.

Stable Histone2B-EGF or Histone2B-mCherry expressing B16-F0 and B16-F10 cell lines were obtained by lentiviral transduction and blasticidin selection (10 μg/mL) and cultured as previously described.[7] Dorsal skin-fold chambers were transplanted on a skin flap of C57/B16 J mice (Charles River) and B16-F0 or B16-F10 Histone2B-EGFP/mCherry cells were implanted by injection of $5x10^4$-$2x10^5$ cells into the dermis adjacent to the deep dermal vascular plexus. For visualization of blood vessels, AlexaFluor750-labeled 70kD dextran was injected intravenously (2 mg/mouse). Before intravital imaging, mice were anesthetized with isofluorane (1-3% in oxygen) and skin-fold chambers were mounted on a temperature-controlled stage (37 ^{o}C).

A customized multiphoton microscope (TriMScope-II, LaVisionBioTec) setup equipped with three tunable Ti-Sa lasers and an optical parametric oscillator was used for intravital imaging. This multiphoton microscopy system allowed for simultaneous second-harmonic generation (SHG) to reconstruct tissue interfaces, and fluorescence imaging to track the movements of B16-F0 and B16-F10 cells stably expressing Histone2B/EGFP into live-mouse dermis.[7] Time-lapse acquisition was done 3-9 days after tumor cell implantation for 1-3 hrs at 5 min intervals. We were able to overcome a number of technical challenges associated with the complexity of both the mouse model and multiphoton microscopy to resolve cell positions and motions with a remarkable precision of ~ 5 μm in the z-direction and into the submicron range for the x-y-directions (see Supplementary Movies S7 and S8 and, for comparison with imaging performed by others, supplemental movies in Refs. [8-12]

All animal experiments were approved by the Ethical Committee on Animal Experiments and performed in the Central Animal Laboratory of the Radboud University, Nijmegen, in accordance with the Dutch Animal Experimentation Act and the European FELASA protocol (www.felasa.eu/guidelines.php).

Reagents for the inhibition and knock-down studies

Drugs. Myosin II inhibitor Blebbistatin was from Sigma-Aldrich (cat# B0560), Rac1 inhibitor NSC23766 was from Tocris Biosciences (cat# 2161), and Arp2/3 inhibitor CKK666 was from Sigma-Aldrich (cat# SML0006).

siRNA. For depletion of actin interacting proteins, following siGENOME SMARTpool siRNAs (Dharmacon) were used: PFN1 SMARTpool siRNAs with target sequences GCAUGGAUCUUCGUACCAA, CCAGAAAUGUUUCGGUGAUC,

GCAAAGACCGGUCAAGUUU, GUGGUUUGAUCAACAAGAA to deplete human Profilin-1 (cat# M-012003-01-0005); CFL1 SMARTpool UGACAGGGAUCAAGCAUGA, GCGGUGCUCUUCUGCCUGA, GUCAAGAUGCUGCCAGAUA, GCUAUGCCCUCUAUGAUGC to deplete human Cofilin-1 (cat# M-012707-00-0005); DIAPH1 SMARTpool GAAGUGAACUGAUGCGUUU, GAAGAGAGAGCAACUCAUA, GGAGAUGGAUGACUUUAAU, GAUAUGAGAGUGCAACUAA to deplete human Dia1 (cat# M-010347-02-0005), and siGENOME Non-targeting siRNA #5 (cat# D-001210-05-05) as control. Cells were transfected with siRNAs at various concentrations (0-100 nM) by using DharmFECT transfection reagent #4 according to the manufacturer's protocol. The efficiency of siRNA transfection was ~96%.

Antibodies and Western blotting. The following monoclonal primary antibodies raised in mouse were used: Cofilin-1 (Santa Cruz, sc-53934), Dia1/DIAPH1 (BD Transduction Laboratories, cat#610848), β-actin (Santa Cruz, sc-47778). In addition, polyclonal antibody raised in goat to Profilin-1 (Santa Cruz, sc-18346) was used. Secondary antibodies were goat anti-mouse conjugated to horseradish peroxidase (HRP) (BD Transduction Laboratories, cat#554002) and donkey anti-goat HRP (Santa Cruz, sc-2033). For immunoblotting, transfected cells were lysed in buffer containing 10 mM Tris-HCl (pH 7.5), 150 mM NaCl, 1% Triton X-100, 1mM EDTA, 10% glycerol and protease inhibitors (Sigma). Protein concentration in lysates was determined by using bicinchoninic acid (BCA) protein assay kit (Pierce). Proteins were separated by SDS-PAGE mini-protean TGX gels (4-15% polyacrylamide) (Bio-Rad), and transferred on PVDF membranes (BioRad). β-actin was used as loading control. Immunoblots were developed by using ECL Western blotting kit (Bio-Rad). Protein depletion was quantified using Image J analysis software.

Data analysis

Heavy-tailed models and fitting procedures. The equations used in the comparisons of the heavy-tailed models can be found in Supplementary Table 1. Power law and exponential distribution parameters were estimated using analytical expression for maximum likelihood estimator, as discussed elsewhere.[13,14] Parameters for truncated power law, log-normal and stretched exponential distributions were found using numerical maximum likelihood estimation and verified by visual inspection of likelihood maps. The upper threshold parameter b for the truncated power law is chosen to be the maximal point in each dataset. The appearance of largely negative μ values in case of log-normal model is demonstrated in Supplementary Figure 6. Effectively, the log-normal curve is being stretched to better fit power-law-like distribution of the data, suggesting that log-normal is not the optimal model for the data. When using only positive μ, the resulting log-normal likelihood and, therefore, wAIC are worse. The resulting parameters are shown in the Supplementary Table 2. Parameter a is a lower cutoff parameter, the choice of which is described below, and b is an upper cutoff parameter used in truncated power law estimation, and corresponds to the maximal point in each dataset.

Lower cutoff estimation. To estimate the choice of the lower cutoff parameter a we used a technique based on reweighted Kolmogorov-Smirnov (rKS) statistic, described in detail in Ref [13]. In brief, the rKS is calculated for all a values taken from the set of unique values of persistence times in each dataset. Then, a is chosen where rKS is minimal and the number of remaining

datapoints after thresholding by *a* is not less than 50% of the original dataset. In cases of truncated and regular power law, exponent values in Figure 2e (main text) are shown for lower cutoffs determined separately using rKS, whereas *w*AIC for all distributions are calculated using cutoff values for the truncated power law.

Outliers/ Data Exclusion. *MDA-MB-231 + NSC data set:* One trajectory was excluded from the analysis shown in Figure 5 because it visually appeared strikingly different from the rest of the cells in this treatment group (n=26 total cell analyzed). Specifically, the excluded cell/ trajectory was (i) much shorter (in time) than other trajectories (<500 min), and (ii) the cell was moving approximately two times faster than rest of the cells in this group. The exclusion did not affect the overall conclusion regarding the motility pattern – motions are superdiffusive and persistence time CFDs fit truncated power law with or without exclusion.

Data and Code Availability

Data and code used to generate results are available from the authors upon request.

Supplementary Note 2. Additional considerations on the fitting bi-exponential distributions

The key difficulty in fitting multi-exponentials is the fact that our data for short/small steps is more "binned", or discretized, than the data described in references[15-17]. The discretization of our data is unavoidable because of short, as well as long-time limitations imposed by slow-moving, rapidly-dividing metastatic cells that also change their shapes as they move. Our data was acquired at one frame per 3 minutes or ~ 320 frames per 16-hr movie (or for long-term trajectories, one frame per 5 min or ~480 frames in 40 h-movies) due to two main considerations: 1) because during $t < 3$-5 min, net translocation of cancer cells is negligible, and 2) to limit photo-damage imposed on cells by frequent laser/light exposure. Unlike humans, animals and bacteria, mammalian cells concurrently with translocation across the substrate continuously change their shape by simultaneously extending membrane protrusions at some regions of their perimeter, while retracting other regions, making the precise detection of cell's position challenging. In order to exclude membrane fluctuations that are not relevant to cell translocation, we have chosen x_{min} ~ 3-5 min, and – instead of analyzing persistence lengths (as has been done in references[15-17]) – have analyzed persistence times. Persistence time is also a parameter that is classically analyzed by researchers in cell motility field as explained in the Introduction section in the main text.

In contrast, works in references[15-17] analyzed trajectories consisting of large number of data points and yielding less discretized cumulative frequency distributions (CFDs) of persistence lengths. Specifically, Raichlen et al. [16] and others who analyzed 2D trajectories of human movements analyzed GPS tracking data which is very detailed, providing precise position information over entire movement paths (for example, Reynolds et al.[18] recorded 950 positions per trajectory). In similar vein, snail movements analyzed by Reynolds et al. [15] were recorded by a

method that visualizes entire movement path. Swarming bacteria (studied by Ariel et al. [17,19]), on the other hand, move much faster than cancer cells –movements were tracked at 100 fps or 6000 frames in 2 min per trajectory – yielding detailed movement trajectories akin those recorded by GPS. Also, the changes of walker's shape are negligible in comparison to most travel distances, hence relative uncertainty of determining the walker's position is much lower than in our experiments with "shape-changing" cells. As a result of the spatial precision, high quality cumulative frequency distributions (CFDs) of persistence lengths could be constructed and analyzed.

Due to the considerations laid out above, admittedly, our datasets have smaller number of datapoints (than those described in [15-17]) and the analysis of persistence times leads to discretized CFDs such that large fraction of data points correspond to $x_{min.}$. While fitting other laws to our data could be performed with reasonable confidence, double-exponential fitting is – infamously – much less stable with respect to noise than single-exponential or power law fits. This, in turn, presents irresolvable difficulties when fitting to the hyperexponential distribution models, as can be illustrated in more detail with the mixture of two exponentials:

$$P(t) = p\lambda_1 e^{-\lambda_1(t-a)} + (1-p)\lambda_2 e^{-\lambda_2(t-a)}$$

The only meaningful way to choose the lower cutoff parameter a is to choose it among the data point values, in particular because a must be the same for all the models compared, and the power law exponent value depends on it strongly. Since, as narrated above, (1) the only feasible way to do long-term cell fluorescent imaging is to take images in time intervals on the order of minutes to avoid the cell damage from light (3 and 5 minutes, in our case), and (2) net translocation of cancer cells over shorter time intervals is negligible, our data is not continuous in time. Data

points for the shortest persistence time interval t_0 are much more numerous than for longer intervals. The choice of a dictates that $t_0 - a = 0$, which makes exponent terms $e^{-\lambda(t_0-a)} = e^0 = 1$ for this interval. Therefore, the problem of likelihood maximization for $t = t_0$ transforms into the maximization of $p\lambda_1 + (1-p)\lambda_2$. It is easy to see that this function grows with either of the exponent parameters $\lambda \to \infty$, which in practice leads to elimination of one of the exponential terms in the mixture. Indeed, all of the numerical methods that we have tried – including Expectation Maximization, known to be particularly suitable for the likelihood maximization of mixtures – lead to an unlimited growth of one of the exponent parameters λ, meaning that hyperexponential functions family are not a suitable modes to fit to our data.

For the reasons narrated above, the fitting of mixtures of exponentials to our data was not possible.

On the other hand, discrete distributions mentioned, for instance, in Ref [13] are developed for working with dimensionless quantities, namely counts. When trying to fit Yule-Simon or Poisson distributions to experimental probability distributions with dimensional units such as minutes in our case, physical interpretation of such models becomes problematic as t here is no clear transformation of these models with scaling of units. Thus, we decided not to consider these models.

Supplementary Note 3. Goodness-of-fit tests

We performed goodness-of-fit tests (see selected example plots below) as described in reference [13]. Briefly, experimental (*blue circles* in Supplementary Figure **7**) and simulated (*green crosses*) distributions of the same sample size were compared to the original best-fit truncated power law distribution (*black curve*) from which the data is sampled during simulation. This method entails determining KS statistic for each comparison and computing corresponding p-value[13]; the latter serves as goodness-of-fit measure of how well the data fits particular model with $p > 0.1$ indicating strong support for the model tested. When goodness-of-fit test is performed according to this method, our experimental data has better KS statistic than simulated data for all data sets (corresponding to Figure 2 of the main text and Supplementary Figure 5) leading to $p = 1$ for PC-3M, B16-F1, MDA-MB-231, MDA-MB-231-long-term data sets (see graphical illustration of goodness-of-fit in Supplementary Figure 7 below).

One caveat of performing goodness-of-fit tests by using the above method is that points for very small persistence times values in simulated CDFs display deviation from the original distribution from which persistence times were drawn (note initial points of curve with *green crosses* deviating from the black curve in Supplementary Figure 7). This deviation is caused by binning of continuous distribution of persistence time values by the frame rate, and is also observed for experimental data.

In order to account for these deviations, we used a complementary approach in which we generated reference truncated power law distribution (*red rectangles*) by generating one million simulated data sets with interval between time points corresponding to experimental image

acquisition frequency (frames per minute, fpm). Using very large sample size effectively reduces the noise to negligible levels. To illustrate the variance of individual simulated data sets, one thousand example data sets (from one million) are shown in shades of grey in Supplementary Figure 7. The comparison of experimental data (*blue circles*) with simulated TP reference distribution (*red rectangles*) provides another way for graphically assessing the goodness-of-fit. Experimental data falling in the middle of the collection of simulated data sets (shown in shades of grey) indicates that experimental data reasonably fits corresponding truncated power law model.

Supplementary Note 4. Analysis of cell trajectories in 3D collagen matrices.

Summary. To address the possibility that Lévy walks derive from the linearity of microfabricated tracks or biological structures (e.g., striated muscles, blood vessels, bundled collagen fibers) in tissues we also analyzed cell motions in a 3D porous lattice model comprising isotropically arranged collagen type I fibrils with cell-permissive gap sizes. This model [5,6] reflects a number of *in vivo* connective tissue-like characteristics (dimensionality, substrate ligands, density, stiffness) but is free of additional extracellular complexity, such as ligand density variation, chemokine deposits and/or cell structures. Experiments and analyses described in Supplementary Figures 8 and 9 **c**onfirm that also in this isotropic ECM-based system, non-metastatic cells (here, MCF-7), move diffusively while metastatic ones (here, MDA-MB-231), perform Lévy walks.

Details of mathematical analysis. Since cells trajectories in gels are curvilinear and distinct cell "steps" cannot be as easily defined as in the case of cells on microtracks, we used analysis adapted from the work of Harris et al. [20] whereby the probability distribution function, $P(r,t)$, was constructed by sampling cell trajectories, $r(t)$, at different time intervals. In doing so, all data was binned such that a constant number of data points were placed in each bin to avoid biasing and to maintain equal statistical error in each bin. The dependencies for each timing interval were then rescaled. A probability distribution function which evolves in time, $P(r,t)$, is said to have scaling symmetry if it can be expressed as $P(r,t) = f(r/t^{\gamma})$, where f is a certain function and γ is a certain number; the probability distributions characterizing both diffusive and Lévy motions have this property. This mathematical characteristic implies that the probability distribution measured at different time intervals should exhibit data collapse – that is, the graphs

of these probability distributions should be transformed onto a single curve upon transformation $r \to \rho = r/t^{\gamma}$. This is, in fact, what we observe for our data shown in Supplementary Figure 9**.** Our analysis reveals that for non-metastatic cells the "collapsed" PDF fits the normal distribution of the form $p(r) = \frac{1}{\sqrt{2\pi\sigma^2}} e^{-\frac{r^2}{\sigma^2}}$ with Akaike weight ~1. In sharp contrast, the metastatic cells have PDFs that fit power law dependence $P(r) = \left(\frac{\mu-1}{r_{\min}}\right)\left(\frac{r}{r_{\min}}\right)^{-\mu}$ with Akaike weights also close to 1. In this case, the power-law exponent, μ ~ 2.3, indicates Lévy walking.

Note: While the overall predictions of the microtrack and the collagen studies are congruent, we note that the analysis of the trajectories in the gel is not as information rich. Specifically, the power law $P(r,t)$ derived as above does not necessarily guarantee the power law in the probability distributions of persistence times (here, to avoid confusion, denoted, $\psi(\Delta t)$) or of the persistence length, $\varphi(\Delta r)$. The probability distribution $P(r,t)$ is related to $\psi(\Delta t)$) and $\varphi(\Delta r)$ through the master equation based on the continuous time random walk model[S2]: $p(r,t) = \delta(r)[1 - \int_0^t \psi(t-t')dt'] + \int_{-\infty}^{\infty} dr' \int_0^{\infty} dt' \phi(r')\psi(t')p(r-r',t-t')$. Note that it is impossible to uniquely determine $\psi(\Delta t)$ and $\varphi(\Delta r)$ for a given $P(r,t)$ because many different cell trajectories, each with its unique $\psi(\Delta t)$ and $\varphi(\Delta r)$, can give rise to the same $P(r,t)$. For example, a cell walking from r to r' at a constant velocity v would have the same $P(r,t)$ as a cell jumping over this distance instantaneously and then "resting" at the final location for a time $(r'-r)/v$. Although the former case is natural and one may consider the latter a special case, what should be emphasized here is that ambiguity is inevitable in the determination of $\psi(\Delta t)$ and $\varphi(\Delta r)$ from $P(r,t)$. We note this ambiguity is absent in the microtrack assays in which the one-dimensional motions of the cell naturally produce $\psi(\Delta t)$ and $\varphi(\Delta r)$ distributions. Therefore, as mentioned in

above text, the two modes of analysis we used are complementary to one another and also to the *in vivo* studies.

Supplementary Note 5. A simple model of Lévy motion resulting from the synchronization of front/back protrusions/retractions.

Model summary. Our previous experiments and computer simulations hinted at a potential relationship between Lévy walks and the synchronization between protrusion/retraction dynamics at the cell-front[21] and at the cell rear[22,23]. Specifically, in reference[24], we studied the protrusion-retraction dynamics in motile metastatic vs. non-metastatic breast cancer cells. Based on the combination of cross-correlation, Granger causality, and morphodynamic profiling analyses we showed[19] that in metastatic cells, the protrusions and retractions are highly "synchronized" both in space and in time; in contrast, protrusions and retractions formed by non-metastatic cells are not "synchronized". In the context of our current work, the relevant question is whether such protrusion-retraction synchronization can give rise to Lévy walking?

To begin with, we considered a stochastic, one-dimensional process in which a material point takes discrete, infinitesimally small steps with some probability "to the right" or "to the left". Based on arguments detailed Section S5.2 below we showed that if the steps are independent of one another (i.e., there is no history and the process is Markovian), the distribution of persistence times always corresponds to a diffusive motion, irrespective of the probabilities of individual steps. We then considered a scenario in which the probabilities of taking left-right steps depend on prior history (non-Markovian process). We showed (see details in Section S5.3) that if consecutive steps slightly favor moves in the "same" direction – e.g., as observed in the microscale dynamics of cell membrane where persistence of membrane protrusion typically

depends on levels of actin regulators, such as Rac1 and Arp2/3, activation of positive feedback loops, precise spatiotemporal regulation of Rho family GTPases and coupling of protrusion to adhesion[20-23]– then the overall distribution $P(n)$ of persistence length n can be a power-law. In particular, if the probability of taking a step in a given direction increases with the number of steps, $k > 1$, already taken in the same direction as

$$q_k = a - b(1 + k)^{-\gamma}, \tag{5.1.1}$$

where $a,\ b,\ \gamma$ are fixed parameters of the model ($0 < a < 1, 0 < b < 2a, \gamma > 0$) then for $a = 1,\ \gamma = 1$ the distribution $P(n)$ at large n is well approximated by a power-law $\propto n^{-b/a-1}$ (see Supplementary Figure 15a). Choosing $a < 1, \gamma = 1$ introduces an exponential cut-off factor a^n in the distribution $P(n)$ of persistence lengths n. This factor becomes significant for $n \gtrsim -1/\ln(a)$ (Supplementary Figure 15b) and it makes the motion pattern resemble random walk. Exponential-like decay of $P(n)$ with n can also be induced by choosing $\gamma < 1$ (Supplementary Figure 15b, grey curve). A detailed analysis of mathematical properties of random process generated by (5.1.1) can be found in subsequent sections.

Naturally, a cell is not a material point and its net motions are determined by the stochastic – and possibly interrelated – displacements of the cell front and cell rear. Accordingly, we considered a model in which a cell was represented by two connected points we call F for front and B for back (Supplementary Figure 16a). The cell's initial length is denoted L_0 and its center of mass is half-way between F and B points. At each discrete step, both the front and the back are allowed to make a step of a certain size (typically, 1/20 of L_0) either "forward" (say, in the $+x$ direction) with probability $q_k = 1-(1+k)^{-\gamma}$ (cf. above) or "backward" ($-x$ direction) with probability $1-q_k$, where k is the number of steps performed in the same direction. After the front

and the back are each allowed to make their individual moves, the position of the cell's center of mass is calculated. Since the cell length can change from step to step and can potentially become unrealistically high, we reject steps in which the cell stretches to more than 3.5-10 times its initial cell lengths (i.e., $L_{\max}$ = 3.5-10 L_0) or contracts more than twice (i.e., $L_{\min}$ = 0.5 L_0; *note*: specific values of $L_{\max}$ and $L_{\min}$ do not alter the general conclusions). The steps are repeated until the cell's center of mass reverses the direction of motion, at which time the step index k is reset to zero. The statistics of such unidirectional excursions are then collected to give rise to probability distributions characterizing the type of motion. We then consider two scenarios:

(1) Full front-back synchronization (Supplementary Figure 16b,d). At a given step, the front and the back are moving forward (+x) or backward (-x) with the same probability (i.e., $q_k = 1 - (1+k)^{-1}$ for forward and $1 - q_k$ for backward). This translates into the front/back moves being fully synchronized in the sense that the front and the back perform the same moves at each time interval – for example, when the tail contracts, the cell's front moves forward. Under these conditions, the cell's center of mass executes a Lévy walk characterized by a power law corresponding to blue markers in Supplementary Figure 16d (slope ~ -2; Akaike weight for the power-law fit > 0.99).

(2) No front-back synchronization (Supplementary Figure 16c,d). The front and back perform independent moves but each with probabilities sampled from the same probability distribution. At each cell step: **(i)** The front is allowed to move in the +x direction with probability $q_k = 1 - (1+k)^{-1}$or in the –x direction with probability $1 - q_{k_{\mathrm{F}}}$ (see Supplementary Figure 16c); **(ii)** The back of the cell is allowed to move in the +x direction with probability $q_k = 1 - (1+k)^{-1}$or in the –x direction with probability $1 - q_{k_{\mathrm{B}}}$ (see Supplementary Figure 16c); **(iii)** If

either the front or the back reverse their direction of motion, their step indices (k_{F} and k_{B}, respectively) are reset to one; **(iv)** The process is repeated until the center of mass reverses direction of motion. As evidenced by the probability distribution in Supplementary Figure 16d (red markers), the case of no front-back synchronization gives rise to a distribution that fits an exponential function, $f(x) = \gamma/e^{\gamma(x-a)}$ with $\gamma \sim 0.73$ and a ~ 11. In this case, the Akaike weight for the exponential vs. power-law fit is ~ 0.75 in favor of the former. It's easy to see that values of parameter $a < 1$ in (5.1.1) resulting in truncation in the Lévy walk described in Section 5.3, have the same effect on fully synchronized Lévy walk.

The general conclusion of the model is therefore that synchronized (or de-synchronized) front-back protrusions/retractions determine the overall motility pattern. The model predicts that cells in which front and back dynamics are synchronized are expected to perform Lévy walks or truncated Lévy walks (as in our experiments with metastatic cells) whereas lack of synchronization should translate into diffusive motion (as in non-metastatic cells). As narrated at the beginning of this Section, these theoretical predictions are in line with our recent work[24] where morphometric analyses of moving metastatic MDA-MB-231 cells evidenced synchronization of the cell-front and cell-back dynamics (with tail retractions directly preceding protrusions of the cell front). Same analyses of motile non-metastatic MCF-7 cells showed that such correlations/synchronization were absent. An open question for future work is what phenomena at the (macro) molecular or cytoskeletal levels are responsible for the synchronization in Lévy walking cells.

Model's mathematical details: A simple Markovian walking with no history. Consider a stochastic process of one-dimensional migration in which the object's trajectory is composed of discrete, infinitesimally small and statistically independent time steps. This is a scenario of a conventional discrete-time random walk and is a Markovian process (see below). We assume that (i) the object continues moving in the same direction if parameter α is greater than some threshold value c, and (ii) reverses the direction of motion if α is less than c. Let us estimate the probability distribution of the total time interval during which the object migrates in the same direction – that is, the distribution of persistence times. Let $p(\alpha)$ denote the probability that at some time the object is characterized by parameter value α (as defined above). Then, at each discrete time step, the probability, q, that the object will maintain its direction of motion is given by $\int_c^\infty p(\alpha)d\alpha = q$ and the probability that it will switch direction of its motion is $\int_0^c p(\alpha)d\alpha = 1 - q$. Using these quantities, we can calculate the probability, $P(n)$, that the moving object persists in one direction but stops at the n-th step (where it reverses its direction of motion). Since the steps are assumed to be independent (Markovian process with no history), $P(n)$ is simply given by $P(n) = q^{n-1}(1-q) = \frac{1-q}{q}q^n$. Because $\frac{1-q}{q}q^n \sim q^n = \exp[\log(q)n]$, we can immediately see that this probability distribution can be approximated as exponential. Note that this exponential behavior does not depend on the functional form of $p(\alpha)$ or the value of c. As long as the function $p(\alpha)$ in a Markovian process is fixed, the probability distribution for persistence times is always an exponential distribution (i.e., the object moves diffusively).

Model's mathematical details: Non-Markoviam walking with history. Next, we consider non-Markovian processes in which the probability of switching the direction of migration decreases as long as unidirectional motion continues. In other words, the longer an object moves in one direction, the (slightly) more likely that it will continue moving in the same direction. Let $p(\alpha, k)$ denote the probability distribution of α at the k-th step before switching the direction of migration. Then, the probability that a unidirectional migration stops at the n-th step, $P(n)$, is given by

$$P(n) = \left(\prod_{k=1}^{n-1} \int_{c}^{\infty} p(\alpha, k) d\alpha\right)\left(\int_{0}^{c} p(\alpha, n) d\alpha\right) = \left(\prod_{k=1}^{n-1} q_k\right)(1 - q_n) \tag{5.3.1}$$

where $q_k = \int_c^{\infty} p(\alpha, k) d\alpha$ is the probability of continuing movement in the same direction after k steps has been made in this direction. In order for the object to be more likely to move in the same direction as it is already moving, q_k has to be a monotonically increasing function with respect to k. Let us calculate the functional forms of $P(n)$ by assuming some specific functions forq_k. For example, when we set $q_k = 1 - \beta \exp(-k/k_0)$ which is independent of α, the probability distribution $P(n)$ becomes exponential, $P(n) \sim \exp\left(-\frac{n}{n_0}\right)$ characterizing a diffusive motion. In the model[24] developed for endosomal transport, approximately exponential saturation of average q_k to a limit of $q_k \approx 0.91$ is the consequence of the underlying equations of the model, although it is unclear whether postulating certain function q_k is equivalent to the full model of [24].

Instead of exponentially saturating q_k, let us consider $q_k = a - b(1+k)^{-\gamma}$, where a, b, γ are fixed parameters of the model. The probability to move forward at the step i after $k = i - 1$ steps forward has already been made is

$$p_i = \begin{cases} 1, & i = 1 \\ a - b/i^{\gamma}, & i > 1 \end{cases} \tag{5.3.2}$$

The convention $p_1 = 1$ reflects that one step in current direction has been made automatically after the preceding turn. This convention simplifies expressions we derive below. We will only consider cases when $\gamma > 0$, so that p_i grows monotonously with i. Since p_i are probabilities, to satisfy the condition $0 < p_i \leq 1, \forall i$ it is necessary that $a \leq 1$ and $b < a \cdot 2^{\gamma}$.

The probability to move forward n steps and then switch direction is

$$P(N = n) = (1 - p_{n+1}) \prod_{i=1}^{n} p_i \tag{5.3.3}$$

We will call $(1 - p_{n+1})$ the "turning probability" factor.

For the case $\gamma = 1$, the asymptotic behavior of $P(N = n)$ for $n \gg 1$ can be evaluated as

$$P_{\gamma=1}(N=n)=(1-p_{n+1})\prod_{i=1}^{n}p_i=(1-p_{n+1})\exp\left(ln\left(\prod_{i=1}^{n}p_i\right)\right)$$

$$=(1-p_{n+1})\exp\left(\sum_{i=1}^{n}ln\,p_i\right)=(1-p_{n+1})\exp\left(\sum_{i=1}^{n}ln\left(a-\frac{b}{i}\right)\right)=$$

$$\approx(1-p_{n+1})\exp\left(\int_1^n ln\left(a-\frac{b}{x}\right)dx\right)=$$

$$=(1-p_{n+1})\exp\left(\left[x\ln\left(a-\frac{b}{x}\right)\right]\Big|_1^n-\int_1^n\frac{x}{a-\frac{b}{x}}\cdot\frac{b\,dx}{x^2}\right)=$$

$$=(1-p_{n+1})\exp\left(\left[x\ln\left(a-\frac{b}{x}\right)\right]\Big|_1^n-\left[\frac{b}{a}\ln(an-b)\right]\Big|_1^n\right)=$$

$$=(1-p_{n+1})\exp\left(n\ln(a-b/n)-\frac{b}{a}\ln(an-b)+\left(\frac{b}{a}-1\right)\ln(a-b)\right)\xrightarrow{n\gg1}$$

$$\xrightarrow{n\gg1}(1-p_{n+1})\exp\left(n\ln\left(a-\frac{b}{n}\right)-\frac{b}{a}\ln(an-b)\right)=a^{n-\frac{b}{a}}n^{-\frac{b}{a}}\left(1-a+\frac{b}{n+1}\right)$$

For $n\gg1$, $a^{n-b/a}\approx a^n$ and $(n+1)\approx n$, so, finally,

$$P_{\gamma=1}(N=n)\approx a^n\cdot n^{-\frac{b}{a}}\cdot\left(1-a+\frac{b}{n}\right)\qquad(5.3.4)$$

First factor in (5.3.4) creates exponential decay $a^n=\exp(n/\ln(a))$, which is approximately $\exp(-n/(1-a))$ if a is close to 1. This factor becomes significant for $n\gtrsim1/(1-a)$. Influence of this factor on $P_{\gamma=1}(N=n)$ for various a is illustrated in Supplementary Figure 15b, where dashed lines show asymptotic formula (5.3.4), while solid lines are calculated by directly evaluating the product (5.3.3). This exponential cut-off in $P(N=n)$ should be present for other saturating functions p_i provided they saturate at some value less than unity. For example, in a

study of endosomal active transport[29], the probability of continuing in current direction saturates at approximately 0.91 (see Fig. 3b in [29]), so one might expect a cutoff in the persistence lengths distribution for persistence lengths larger than about $1/\ln(0.91) \approx 10$ steps. However, no such cutoff is visible on simulated distribution shown on Fig. 3c in [29]. This discrepancy is most likely due to the fact that in reference [29] the probability p_i is not postulated, but emerges as a consequence of intrinsic model equations: model in [29] is a complex random process tracking such internal variables as number of motors attached to the cargo at a given time, and is not equivalent to a random process that decides the direction of next step based solely on the number of steps made in the current direction.

Second factor in (5.3.4) is the power law $n^{-b/a}$. But for $n < b/(1-a)$ the term $\left(1 - a + \frac{b}{n}\right)$ is dominated by b/n, which adds (-1) to the overall exponent and expression (5.3.4) scales as $n^{-b/a-1}$ rather than $n^{-b/a}$. For example, for $\{a = 1,\ b = 1,\ \gamma = 1\}$, $P_{\gamma=1}(N = n) \xrightarrow{n\to\infty} n^{-2}$ (dark blue line in Supplementary Figure 15a and bright yellow line in Supplementary Figure 15b). For $\{a = 1,\ \gamma = 1\}$ and $b = 0.5, 1.0, 1.5, 1.99$ the exact product (5.3.3) is shown in Supplementary Figure 15a – for large n it reaches asymptotic power laws $n^{-b/a-1}$ as indicated on the graph.

Setting $\gamma < 1$ results in decay of $P(n)$ (Supplementary Figure 15a, grey solid line) that can be approximated as exponential (Supplementary Figure 15a, grey dashed line). In other words, to drive this model closer to random walk behavior, using the values $\{a = 1,\ \gamma < 1\}$ is an alternative to using the values $\{a < 1, \gamma = 1\}$ discussed above.

Since this is a discrete model, fitting it to experimental data faces the difficulty of determining the length of the step in the units of time, as discussed in Supplementary Note 2. As can be seen by, e.g. trying to rescale n in asymptotic formula (5.3.4), there is no clear way to rescale parameters a, b, γ when the time scale is changed, thus the length of the step should be viewed as an additional unknown parameter. Due to this ambiguity of the step length, we do not include this model in the goodness-of-fit tests performed above. Here, we show fitting of this model to CFDs for MDAMB231 long-term dataset to illustrate how the model's exponential cut-off of a Lévy walk compares to truncated Lévy walk model used in Supplementary Figure 5. For this purpose, we somewhat arbitrarily chose step length equal to the experimental time bin (5 min) and assumed that the first experimental point ($t = 10\,\text{min}$) corresponds to $n = 2$ of the discrete model. The best fit of the model with fixed $\gamma = 1$ (Supplementary Figure 15e, blue curve) was somewhat inferior to the best fit when all three model parameters were varied (Supplementary Figure 15e , green solid curve). The best fit of Truncated Lévy Walk obtained in Supplementary Figure 5 and Supplementary Tables 2 and 3 for this dataset is shown by the black curve on Supplementary Figure 15e for comparison.

Now we will briefly discuss the model's behavior for $\gamma > 1$. If $a = 1$ and $\gamma > 1$, there is a finite probability that cell will never turn and instead continue walking in one direction forever (this is proved as a theorem below). Probability of such infinite walk is plotted on Supplementary Figure 15c. For example, for $\{a = 1, b = 1, \gamma = 2\}$, about half of the cells will never change their direction of travel. But those cells that happen to not walk forever in one direction have a distribution of persistence lengths that behaves as a power law. The exponent of this power law has a non-monotonous dependence on γ. For $\gamma = 1$, as discussed above, the "turning probability"

in (5.3.3) is $\propto n^{-1}$ which adds to the in the $\prod_{i=1}^{n} p_i \propto n^{-1}$ and results in overall power law $\propto n^{-2}$. For $\gamma > 1$, factor $\prod_{i=1}^{n} p_i$ converges to some slowly decaying asymptote (we don't provide a rigorous proof of this statement) and the decay of $P(n)$ mostly comes from the "turning probability" factor $\propto n^{-\gamma}$. For this reason, $P(n) \propto n^{-2}$ both for $\gamma = 1$ and $\gamma = 2$ (Supplementary Figure 15d).

Theorem 5.3.1: Probability of walking in the same direction forever during a walk generated by (5.3.2) is non-zero for $a = 1, \gamma > 1$ and zero otherwise.

Proof. Cumulative probability to walk forward m steps or less is

$$P(N \le m) = \sum_{n=1}^{m} P(N = n) = \sum_{n=1}^{m} \left[(1 - p_{n+1}) \prod_{i=1}^{n} p_i \right] = \\ = \sum_{n=1}^{m} \left[\prod_{i=1}^{n} p_i - \prod_{i=1}^{n+1} p_i \right] = \sum_{n=1}^{m} \prod_{i=1}^{n} p_i - \sum_{n=1}^{m} \prod_{i=1}^{n+1} p_i = 1 - \prod_{i=1}^{m+1} p_i \tag{5.3.5}$$

For evaluating cumulative probability $P(N \le \infty)$ we can use limit comparison test: if $a_n \xrightarrow{n\to\infty} 0$, then from well-known limit

$$\lim_{n\to\infty} \frac{\ln(1 + a_n)}{a_n} = 1 \tag{5.3.6}$$

and the limit comparison test it follows that infinite product $\prod_{n=1}^{\infty}(1 + a_n)$ converges if and only if the sum $\sum_{n=1}^{\infty} a_n$ converges. Now we can evaluate

$$P(N \le m) = 1 - \prod_{i=1}^{m+1} p_i = 1 - \prod_{i=1}^{m+1} \left(a - \frac{b}{i^\gamma} \right) = 1 - \prod_{i=1}^{m+1} \left(1 - \left((1 - a) + \frac{b}{i^\gamma} \right) \right)$$

If the sum

$$\sum_{i=1}^{\infty} \left((1 - a) + \frac{b}{i^\gamma} \right) \tag{5.3.7}$$

converges to a finite positive value, then $0 < P(N \le \infty) < 1$, which means that there is a finite probability of infinite walk in one direction. But if this sum diverges, then

$$\prod_{i=1}^{m+1}\left(1-\left((1-\ a)+\frac{b}{i^{\gamma}}\right)\right)\xrightarrow{m\to\infty}0 \tag{5.3.8}$$

and therefore $P(N \leq \infty) = 1$, which means that infinitely long walks are impossible. It's easy to see that

$$\sum_{i=1}^{\infty}\left((1-a)+\frac{b}{i^{\gamma}}\right)=b\zeta(\gamma)+\sum_{i=1}^{\infty}(1-a) \tag{5.3.9}$$

where $\zeta(s)$ is the Riemann zeta function

$$\zeta(s) = \sum_{k=1}^{\infty}\frac{1}{k^{s}}$$

which converges for $s > 1$ and diverges for $s \leq 1$. Obviously, the sum $\sum_{i=1}^{\infty}(1-a)$ converges only if $a = 1$. Therefore, infinite walks in one direction are possible if $\gamma > 1, a = 1$ and are impossible for all other values of a and γ.

Supplementary Tables

Distribution	Probability density function *P(t)*
Power law	$t^{-\mu}\frac{\mu-1}{a^{1-\mu}}$
Truncated power law	$t^{-\mu}\frac{\mu-1}{a^{1-\mu}-b^{1-\mu}}$
Log-normal	$\frac{1}{t}exp\left[\frac{-(lnt-\mu)^2}{2\sigma^2}\right]\sqrt{\frac{2}{\pi\sigma^2}}\left[erfc\left(\frac{a-\mu}{\sqrt{2}\sigma}\right)\right]^{-1}$
Stretched exponential	$t^{\beta-1}e^{\lambda(a^{\beta}-t^{\beta})}\beta\lambda$

Supplementary Table 1. Distributions used for comparing heavy-tailed models. Parameters *a* and *b* are lower and upper cut-offs, respectively.

Cell type	*a, min*	Truncated power law	Power law	Log-normal	Stretched exponential
MDAMB231 #1(WETS)	6	μ = 2.49	μ = 2.55	μ = -19.78, σ = 3.8329	λ = 20.95, β = 0.0636
MDAMB231 #2 /Long (CYTOO)	10	μ = 2.82	μ = 2.84	μ = -797.73, σ = 20.8856	λ = 240.7511, β = 0.0075
PC-3M	9	μ = 2.22	μ = 2.48	μ = 1.6829, σ = 1.0506	λ = 0.4998, β = 0.5795
B16-F1	6	μ = 2.99	μ = 3.01	μ = -731.41, σ = 19.1417	λ = 273.46, β = 0.0072
MCF-7	6	μ = 4.52	μ = 4.57	μ = -409.76, σ = 10.7604	λ = 705.93, β = 0.005
PC-3	6	μ = 3.14	μ = 3.25	μ = -647.41, σ = 17.0115	λ = 172.92, β = 0.0126
B16-F0	3	μ = 3.69	μ = 3.71	μ = -535.57, σ = 14.075	λ = 709.21, β = 0.0038

Supplementary Table 2. Parameters for heavy-tailed distribution models. All parameters were obtained using the lower cutoff value a corresponding to truncated power law and also shown here and in Table 1 in the main text.

Cell type	Speed (*μm/min)*	α (MSD)	Lévy exponent *μ*	TP	P	LN	SE	E
MDA231 #2 /Long (CYTOO)	1.13	1.46	2.81	**>0.65**	<0.01	0.34	<0.01	<0.01
MDA231 #1 (WETS)	0.99	1.54	2.49	**>0.99**	<0.01	<0.01	<0.01	<0.01

Supplementary Table 3. Model parameters and Akaike weights (*w*AIC) for MDA-MB-231 cells on WETS vs. CYTOO 1D microtrack substrates. WETS data set (MDA231 #1 WETS, n= 69 cells) is described and analyzed in Figures 1, 2 and 5 in the main text. CYTOO long-trajectory data set (MDA231 #2/Long CYTOO; n=49 cells) is described in Supplementary Figures 2 and 5. Cell mean speed, exponent α (from MSD) and Lévy exponent μ (from fitting truncated power law) and Akaike information criteria weights (wAIC) for model comparisons. TP = power law, power law = P, log-normal = LN, stretched exponential = SE and exponential = E distributions. Strongest supported model is highlighted in bold. Cell motions are superdiffusive and truncated power law is favored for both data sets/substrates. See also Supplementary Figures 2 and 5 for trajectory/displacement, MSD vs. time, cumulative frequency distribution plots and details on model fitting.

	α	**Mean Speed** ***[μm/min]***	**Median Speed** ***[μm/min]***
**B16F0**	1.05	0.41	0.24
**B16F1**	1.52	0.62	0.49
**PC3**	1.04	0.71	0.49
**PC3M**	1.58	1.13	0.99
**MCF7**	0.96	0.58	0.49
**MDAMB231 #1**	1.54	0.99	0.73
**Blebb 10uM**	1.83	1.13	0.98
**Blebb 50uM**	1.70	0.54	0.49
**NSC 50uM**	1.50	0.86	0.73
**NSC 100uM**	1.51	0.64	0.49
**NSC100+Blebb10**	1.07	0.42	0.24
**CK666**	1.01	0.57	0.49
**MDAMB231 #2**	1.46	1.13	0.84

Supplementary Table 4. Summary of cell motility parameters for cells on 1D microtracks. Summary of cell mean speed and α exponent values for cell populations analyzed in the main text. The distributions of cell speeds are shown in Supplementary Figures 3 and 12, and MSD vs. time plots are shown in main text Figures 2 and 5. MDA-MB-231 #1 corresponds to data set analyzed in Figures 2 and 5, and MDA-MB-231 #2 corresponds to long-term data set (total observation time = 40h) analyzed in Supplementary Figure 5 and Supplementary Table 3.

Supplementary References

1. Kandere-Grzybowska, K., Campbell, C., Komarova, Y., Grzybowski, B.A. & Borisy, G.G. Molecular dynamics imaging in micropatterned living cells. *Nat. Methods* **2**, 739-741 (2005).
2. Kandere-Grzybowska, K., *et al.* Cell motility on micropatterned treadmills and tracks. *Soft Matter* **3**, 672-679 (2007).
3. Mahmud, G., *et al.* Directing cell motions on micropatterned ratchets. *Nat. Phys.* **5**, 606-612 (2009).
4. Langhofer, M., Hopkinson, S.B. & Jones, J.C.R. The matrix secreted by 804g cells contains laminin-related components that participate in hemidesmosome assembly in-vitro. *J. Cell Sci.* **105**, 753-764 (1993).
5. Wolf, K. & Friedl, P. Extracellular matrix determinants of proteolytic and non-proteolytic cell migration. *Trends Cell Biol.* **21**, 736-744 (2011).
6. Wolf, K., *et al.* Compensation mechanism in tumor cell migration: mesenchymal-amoeboid transition after blocking of pericellular proteolysis. *J. Cell Biol.* **160**, 267-277 (2003).
7. Weigelin, B., Bakker, G.-J. & Friedl, P. Intravital third harmonic generation microscopy of collective melanoma cell invasion. *IntraVital* **1**, 32043 (2012).
8. Kienast, Y., *et al.* Real-time imaging reveals the single steps of brain metastasis formation. *Nat. Med.* **16**, 116-122 (2010).
9. Kedrin, D., *et al.* Intravital imaging of metastatic behavior through a mammary imaging window. *Nat. Methods* **5**, 1019-1021 (2008).
10. Tozluoglu, M., *et al.* Matrix geometry determines optimal cancer cell migration strategy and modulates response to interventions. *Nat. Cell Biol.* **15**, 751-762 (2013).
11. Manning, C.S., Hooper, S. & Sahai, E.A. Intravital imaging of SRF and Notch signalling identifies a key role for EZH2 in invasive melanoma cells. *Oncogene* **34**, 4320-4332 (2015).
12. Condeelis, J. & Segall, J.E. Intravital imaging of cell movement in tumours. *Nat. Rev. Cancer* **3**, 921-930 (2003).
13. Clauset, A., Shalizi, C.R. & Newman, M.E.J. Power-law distributions in empirical data. *SIAM Review* **51**, 661-703 (2009).
14. Edwards, A.M., *et al.* Revisiting Lévy flight search patterns of wandering albatrosses, bumblebees and deer. *Nature* **449**, 1044-1048 (2007).
15. Reynolds, A., Santini, G., Chelazzi, G. & Focardi, S. The Weierstrassian movement patterns of snails. *R. Soc. Open Sci.* **4**, 160941 (2017).
16. Raichlen, D.A., *et al.* Evidence of Lévy walk foraging patterns in human hunter-gatherers. *Proc. Natl. Acad. Sci. U S A* **111**, 728-733 (2014).
17. Ariel, G., *et al.* Swarming bacteria migrate by Lévy walk *Nat. Commun.* **6**, 8396 (2015).
18. Reynolds, A., Ceccon, E., Baldauf, C., Karina Medeiros, T. & Miramontes, O. Levy foraging patterns of rural humans. *PLoS One* **13**, e0199099 (2018).
19. Ariel, G., Be'er, A. & Reynolds, A. Chaotic model for Lévy walks in swarming bacteria. *Phys. Rev. Lett.* **118**, 228102 (2017).

20. Harris, T.H., *et al.* Generalized Lévy walks and the role of chemokines in migration of effector CD8(+) T cells. *Nature* **486**, 545-548 (2012).
21. Mogilner, A. & Oster, G. Force generation by actin polymerization II: the elastic ratchet and tethered filaments. *Biophys. J.* **84**, 1591-1605 (2003).
22. Yanagida, T. & Ishijima, A. Forces and steps generated by single myosin molecules. *Biophys. J.* **68**, 312S-318S; discussion 318S-320S (1995).
23. Ruegg, C., *et al.* Molecular motors: force and movement generated by single myosin II molecules. *News Physiol. Sci.* **17**, 213-218 (2002).
24. Hermans, T.M., *et al.* Motility efficiency and spatiotemporal synchronization in non-metastatic vs. metastatic breast cancer cells. *Integr. Biol. (Camb.)* **5**, 1464-1473 (2013).
25. Machacek, M. & Danuser, G. Morphodynamic profiling of protrusion phenotypes. *Biophys. J.* **90**, 1439-1452 (2006).
26. Machacek, M., *et al.* Coordination of Rho GTPase activities during cell protrusion. *Nature* **461**, 99-103 (2009).
27. Mendoza, M.C., Vilela, M., Juarez, J.E., Blenis, J. & Danuser, G. ERK reinforces actin polymerization to power persistent edge protrusion during motility. *Sci. Signal.* **8**, ra47 (2015).
28. Castro-Castro, A., *et al.* Coronin 1A promotes a cytoskeletal-based feedback loop that facilitates Rac1 translocation and activation. *EMBO J.* **30**, 3913-3927 (2011).
29. Chen, K.J., Wang, B. & Granick, S. Memoryless self-reinforcing directionality in endosomal active transport within living cells. *Nat. Mater.* **14**, 589-593 (2015).
