# Supplementary material for: Lévy-like movement patterns of metastatic cancer cells revealed in microfabricated systems and implicated in vivo
Source: Nat Commun. 2018 Oct 31;9:4539. doi: 10.1038/s41467-018-06563-w (PMC6208440; doi:10.1038/s41467-018-06563-w)
Supplement: Supplementary file 2 — Description of Additional Supplementary Files [file 41467_2018_6563_MOESM2_ESM.pdf]

Description of Additional Supplementary Files

File Name: Supplementary Movie 1

Description: Lévy walks of invasive MDA-MB-231 cells on linear microtracks (Track width, 20 μm; Movie length, 13 hr). See representative trajectories in Fig. 1.

File Name: Supplementary Movie 2

Description: Diffusive walks of non-invasive MCF-7 cells on linear microtracks (Track width, 20 μm; Movie length, 16 hr). See representative trajectories in Fig. 1.

File Name: Supplementary Movie 3

Description: Lévy walks of invasive PC-3M prostate cancer cells on linear microtracks (Track width, 20 μm; Movie length, 10 hr). See representative trajectories in Supplementary Fig. 1.

File Name: Supplementary Movie 4

Description: Diffusive walks of non-invasive PC-3 prostate cancer cells on linear microtracks (Track width, 20 μm; Movie length, 16 hr). See representative trajectories in Supplementary Fig.1.

File Name: Supplementary Movie 5

Description: Lévy walks of metastatic B16-F1 cells on linear microtracks (Track width, 20 μm; Movie length, 13 hr). See representative trajectories in Supplementary Fig. 1.

File Name: Supplementary Movie 6

Description: Diffusive walks of non-metastatic B16-F0 cells on linear microtracks (Track width, 20 μm; Movie length, 13 hr). See representative trajectories in Supplementary Fig. 1.

File Name: Supplementary Movie 7

Description: B16-F0 melanoma cells (cell nuclei marked with Histone-2B-mCherry are shown in *green*) moving inside of the skin of a live mouse (blood vessels, *red*; collagen, *blue*) as

imaged 7 days after tumor implantation. Bottom panel shows cell trajectories. See also Fig. 3 and 4.

File Name: Supplementary Movie 8

Movie S8: B16-F10 melanoma cells (cell nuclei marked with Histone-2B-mCherry are shown in *green*) invading into the dermis of a live mouse imaged 7 days after tumor implantation (blood vessels, *red*; collagen, *blue*). Bottom panel shows cell trajectories. See also Fig. 3 and 4.

File Name: Supplementary Movie 9

Description: MDA-MB-231 cells reverted to diffusive walkers upon treatment with Arp 2/3 inhibitor CK666 (Track width, 20 μm; Movie length, 16 hr). See representative trajectories in Fig. 5.

File Name: Supplementary Movie 10

Description: MDA-MB-231 cells rendered ballistic movers upon treatment with Myosin II inhibitor Blebbistatin (Track width, 20 μm; Movie length, 12 hr). See representative trajectories in Fig. 5.

File Name: Supplementary Movie 11

Description: Lévy walks of MDA-MB-231 cells moving on microtracks in the presence of Rac1 inhibitor NSC23766. (Track width, 20 μm; Movie length, 8.5 hr). See representative trajectories in Fig. 5.

File Name: Supplementary Movie 12

Description: MDA-MB-231 cells reverted to diffusive walkers upon simultaneous treatment with Blebbistatin and NSC23766 (Track width, 20 μm; Movie length, 16 hr). See representative trajectories in Supplementary Fig. 11.

File Name: Supplementary Movie 13

Description: Movie showing representative trajectory corresponding to Lévy walks of invasive MDA-MB-231 cells on linear microtracks (track width, 20 μm). Cells were labeled with Cell

Tracker Green (shown in blue), speed-coded trajectory is shown in color. See representative long-term trajectories in Supplementary Fig. 2.

File Name: Supplementary Movie 14

Description: Movie showing second example of trajectory corresponding to Lévy walks of invasive MDA-MB-231 cells on linear microtracks (track width, 20 μm). Cells were labeled with Cell Tracker Green (shown in blue), speed-coded trajectory is shown in color. See representative long-term trajectories in Supplementary Fig. 2.

File Name: Supplementary Movie 15

Description: Movie showing third example of trajectory corresponding to Lévy walks of invasive MDA-MB-231 cells on linear microtracks (track width, 20 μm). Cells were labeled with Cell Tracker Green (shown in blue), speed-coded trajectory is shown in color. See representative long-term trajectories in Supplementary Fig. 2.

File Name: Supplementary Movie 16

Description: Protrusion dynamics in control MDA-MB-231 cells corresponding to time series images shown in Supplementary Fig. 14.

File Name: Supplementary Movie 17

Description: Protrusion dynamics in MDA-MB-231 cells treated with CK666 and corresponding to time series images shown in Supplementary Fig. 14.

File Name: Supplementary Movie 18

Description: Protrusion dynamics in MDA-MB-231 cells treated with NSC23766 and Blebbistatin and corresponding to time series images shown in Supplementary Fig. 14.
